# Unrooted unordered homeomorphic subtree alignment of RNA trees

**DOI:** 10.1186/1748-7188-8-13

**Published:** 2013-04-16

**Authors:** Nimrod Milo, Shay Zakov, Erez Katzenelson, Eitan Bachmat, Yefim Dinitz, Michal Ziv-Ukelson

**Affiliations:** 1 Department of Computer Science, Ben-Gurion University of the Negev, Beer-Sheva, Israel; 2 Department of Computer Science and Engineering, UC San Diego, La Jolla, CA, USA

**Keywords:** RNA structure, Tree alignment, Homeomorphic subtree alignment, Unrooted, algorithm, Cavity matching

## Abstract

We generalize some current approaches for RNA tree alignment, which are traditionally confined to *ordered rooted* mappings, to also consider *unordered unrooted* mappings. We define the *Homeomorphic Subtree Alignment* problem (*HSA*), and present a new algorithm which applies to several modes, combining global or local, ordered or unordered, and rooted or unrooted tree alignments. Our algorithm generalizes previous algorithms that either solved the problem in an asymmetric manner, or were restricted to the rooted and/or ordered cases. Focusing here on the most general unrooted unordered case, we show that for input trees *T* and *S*, our algorithm has an *O*(*n*_*T*_*n*_*S*_ + min(*d*_*T*_,*d*_*S*_)*L*_*T*_*L*_*S*_) time complexity, where *n*_*T*_,*L*_*T*_ and *d*_*T*_ are the number of nodes, the number of leaves, and the maximum node degree in *T*, respectively (satisfying *d*_*T*_ ≤ *L*_*T*_ ≤ *n*_*T*_), and similarly for *n*_*S*_,*L*_*S*_ and *d*_*S*_ with respect to the tree *S*. This improves the time complexity of previous algorithms for less general variants of the problem.

In order to obtain this time bound for HSA, we developed new algorithms for a generalized variant of the *Min-Cost Bipartite Matching* problem (*MCM*), as well as to two derivatives of this problem, entitled *All-Cavity-MCM* and *All-Pairs-Cavity-MCM*. For two input sets of size *n* and *m*, where *n* ≤ *m*, *MCM* and both its cavity derivatives are solved in *O*(*n*^3^ + *nm*) time, without the usage of priority queues (e.g. Fibonacci heaps) or other complex data structures. This gives the first cubic time algorithm for *All*-*Pairs*-*Cavity*-*MCM*, and improves the running times of *MCM* and *All*-*Cavity*-*MCM* problems in the unbalanced case where *n* ≪ *m*.

We implemented the algorithm (in all modes mentioned above) as a graphical software tool which computes and displays similarities between secondary structures of RNA given as input, and employed it to a preliminary experiment in which we ran all-against-all inter-family pairwise alignments of RNAse P and Hammerhead RNA family members, exposing new similarities which could not be detected by the traditional rooted ordered alignment approaches. The results demonstrate that our approach can be used to expose structural similarity between some RNAs with higher sensitivity than the traditional *rooted ordered* alignment approaches. Source code and web-interface for our tool can be found in http://www.cs.bgu.ac.il/\~negevcb/FRUUT.

## Background

Secondary structure of RNA molecules serves important functions in many non-coding RNAs [1]. Functional constraints lead to evolutionary structural conservation that in many cases exceeds the level of sequence conservation. Thus, detecting similarity between RNA secondary structures is of major importance in functional RNA research [2,3].

A mainstream approach for (pseudoknot free) RNA secondary structure comparison represents them as trees, and applies tree alignment algorithms [4-6] to their comparison.

Several variants of tree edit distance and alignment problems were previously studied. These variants differ in the type of trees they examine (ordered/unordered, rooted/unrooted), in the type of edit operations or alignment restrictions they apply [4-14], and in their algorithmic approaches (see [7]).

**Figure 1:**
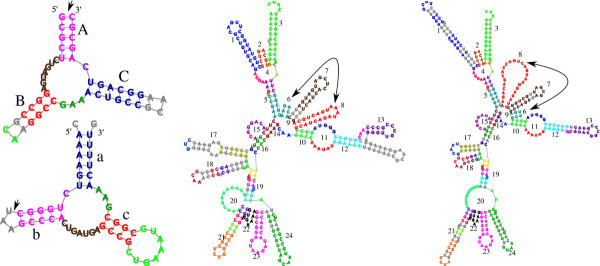
**Unrooted and unordered RNA similarities.** Nodes of the RNA trees are clustered to motifs marked by letters or numbers (stems, loops, and unpaired nucleotide intervals), where aligned motifs share the same annotation, and unaligned nodes are in gray. Nomenclature is according to [50]. (**a**) An unrooted alignment between Hammerhead RNAs: *PDB_00693* (Type I, top) and *RFA_00388* (Type III, bottom), with a computed and corrected, p-value of 2.1250 × 10^-7^. Arrows mark the roots chosen by our tool. The unrooted mode of FRUUT identifies the high similarity between the molecules, not being restricted to align external loops to each other. (**b**) An unordered alignment between RNAse P RNAs: *ASE_00047* (left) and *ASE_00334* (right), with a computed, and corrected, p-value of 1.190 × 10^-4^. In the unordered mode of FRUUT, the aligned motifs marked by 6 and 8 do not preserve order. In both molecules, pseudoknots occur between intervals annotated by 8 and 2, and between intervals annotated by 13 and 15 (see Figure 12), asserting the validity of the alignment.

Currently available bioinformatic softwares for RNA tree comparison usually apply *rooted ordered tree alignment*[11,12,14,15]. However, there are known evolutionary phenomena, such as segment insertions, translocations and reversals, which may result in a reordering or re-rooting of RNA structural elements [16]. These events can yield two similarly structured motifs, which are rooted differently (with respect to the standard “external loop” corresponding roots) [17], and/or permuted with respect to branching order. There are known examples of such unrooted/unordered RNA structural conservations [18,19] (Figure 1), therefore, it is possible that searching for unordered and unrooted structural similarities may reveal new relations between RNA molecules that were previously undetected.

The general *Unordered Tree Edit Distance* problem is MAX-SNP hard [20], promoting the study of constrained variants. The *Subtree Isomorphism* problem [21,22] is, given a pattern tree *S* and a text tree *T*, to find if there is some subtree *T*′ of *T* which is isomorphic to *S*. The *Subtree Homeomorphism* problem [23-25] is a variant of the former problem, where degree-2 nodes may be deleted from the selected subtree *T*′ of the text. Pinter et al. [26] efficiently solved the Subtree Homeomorphism problem, under the unrooted unordered settings. In addition, their algorithm assigns costs for alignments and finds an alignment of minimum cost, thus solving a weighted variant of the problem. The running time of the algorithm of [26] is $O(n_{S}^{2}n_{T}+n_{S}n_{T}\log n_{T})$, where *n*_*T*_ and *n*_*S*_ are the number of nodes in *T* and *S*, respectively (improved time complexities under some scoring scheme restrictions were also shown in [26]). The *Constrained Edit Distance Between Unordered Labeled Trees* problem, presented by Zhang in [27], is a restricted version of rooted unordered tree edit distance, which allows the edit operations of node relabeling, subtree pruning, and deletions of degree-2 nodes (where in the general edit distance variant, nodes of arbitrary degrees may be deleted). Zhang gave an *O*(*n*_*T*_*n*_*S*_(*d*_*T*_ + *d*_*S*_)*log*(*d*_*T*_ + *d*_*S*_)) time algorithm for this variant, where *d*_*T*_ and *d*_*S*_ are the maximum node degrees in *T* and *S* respectively. In this sense, the algorithm of [27] can be viewed as a symmetric (allowing deletions from both input trees), yet rooted variant of the algorithm of [26].

The essential approach in many tree comparison algorithms is a recursive rooted comparison of subtrees of the input trees, and finding the best combination of such sub-instance solutions to yield a solution for the input instance. The computation considers the cases in which either one of the roots is deleted, and the case where the roots are aligned to each other. In the latter case, it is required to find an optimal matching between the two children sets of the roots, where in the ordered variant such matching is restricted to maintain the child order (and may be computed by a reduction to sequence alignment), and in the unordered variant no such restriction holds (and thus an optimal matching can be found by a bipartite graph matching algorithm).

### Our contribution

We propose an efficient algorithm for comparing unordered unrooted trees. Specifically, we define the *Homeomorphic Subtree Alignment* (HSA) problem, for which we give an *O*(*n*_*T*_*n*_*S*_ + *min*(*d*_*T*_,*d*_*S*_)*L*_*T*_*L*_*S*_) running time algorithm, where *L*_*T*_ and *L*_*S*_ are the numbers of leaves in the input trees *T* and *S*, respectively. Our approach can be viewed as a generalization of the two previous works [26,27], which relaxes the asymmetric “text-pattern” restriction of [26], as well as the rooting restriction of [27].

Both algorithms in [26,27], as well as the algorithm presented here, make use of subroutines for solving the *Minimum Cost Bipartite Matching* (*MCM*) problem, which dictate their time complexities. Here, we define the *All*-*Pairs*-*Cavity*-*MCM* problem, a generalization of the *All*-*Cavity*-*MCM* problem [28], and show how to integrate it into our tree alignment algorithm. For *MCM* and both its cavity derivatives, we use similar ideas to those applied in [29] to obtain *O*(*n*^3^ + *nm*) time algorithms, where *n* and *m* are the sizes of the input sets, and *n* ≤ *m*. This gives the first cubic time algorithm for *All*-*Pairs*-*Cavity*-*MCM*, and improves the running times of *MCM* and *All*-*Cavity*-*MCM* problems in the unbalanced case where *n* ≪ *m*. The new *MCM* algorithms we developed allow our HSA algorithm to match, and even improve, the running times of the previous algorithms of [26,27] for less general variants of the HSA problem.

We implemented the algorithm (in all combininations of global or local, ordered or unordered, and rooted or unrooted modes) as a graphical software tool named *FRUUT* which computes and displays similarities between secondary structures of RNA given as input, and employed it to a preliminary experiment in which we ran all-against-all inter-family pairwise alignments of RNAse P and Hammerhead RNA family members, exposing new similarities which could not be detected by the traditional rooted ordered alignment approaches. The results demonstrate that our approach can be used to expose structural similarity between some RNAs with higher sensitivity than the traditional *rooted ordered* alignment approaches.

## Preliminaries

### Tree notations

A *treeT* = (*V*,*E*) is an undirected, connected and acyclic graph. For a node *v* ∈ *V*, denote by *N*(*v*) the set of *neighbors* of *v*: *N*(*v*) = {*u* ∈ *V*:(*v*,*u*) ∈ *E*}. Denote by *d*_*v*_ = |*N*(*v*)| the *degree* of *v*. A node *v* for which *d*_*v*_ ≤ 1 is called a *leaf* in *T*. For simplicity, we henceforth use the notation *v* ∈ *T* and (*v*,*u*) ∈ *T* to imply that *v* is a node and (*v*,*u*) is an edge in a tree *T*. We use the notation (*v* → *u*) to indicate that the generally undirected edge (*v*,*u*) is being considered with respect to the specific direction from *v* to *u*. Denote by *n*_*T*_, *L*_*T*_, and *d*_*T*_ the number of nodes, the number of leaves, and the maximum degree of a node in *T*, respectively.

A *rooted tree* is a tree in which one of the nodes is selected as its *root*. Denote by *T*^*v*^ the tree *T* when rooted upon the node *v* ∈ *T*. An *ordered* tree is a tree *T* in which for each node *v* ∈ *T*, the elements in *N*(*v*) are ordered. In this work we consider *unrooted unordered* trees, *rooted unordered* trees, *unrooted ordered* trees, and *rooted ordered* trees. If no indication is given, we assume that the mentioned trees are unrooted and unordered.

Let *T* = (*V*,*E*) be a tree. A *smoothing of a nodev* of degree 2 in *T* is obtained by removing *v* from *T* and connecting its two neighbors by an edge. A *smoothing* of *T* is a tree obtained by smoothing zero or more nodes in *T*. A *subtree* of *T* is a connected subgraph of *T*. For an edge (*v* → *u*) ∈ *T*, denote by $T_{u}^{v}$ the *rooted subtree* of *T* induced by *v* as a root, and all nodes *x* in *T* such that the path between *v* and *x* in *T* starts with (*v* → *u*).

Since a tree *T* with *n* nodes contains *n* - 1 undirected edges, the total number of directed edges, and hence the number of rooted subtrees of the form $T_{u}^{v}$, is 2(*n* - 1).

A *pruning* of a tree *T* with respect to an edge (*v* → *u*) is the removal from *T* of all nodes in $T_{u}^{v}$, except for *v*. Observe that every nonempty subtree of *T* is obtained by pruning *T* with respect to zero or more edges.

### Min-Cost bipartite matching

Similarly to previous tree alignment and edit distance algorithms [26-28], the algorithm presented here makes use of min-cost bipartite matching algorithms as subroutines. Below, we define extended variants of the bipartite matching problem, in which the input groups may be ordered or unordered, and the score incorporates both standard element matching scoring terms, as well as penalties for unmatched elements. In addition, we define “cavity” variants of the problem, which are used for speeding up our tree alignment algorithms.

#### *The (generalized) Min-Cost bipartite matching problem (MCM)*

Let *X* and *Y* be two sets. A *bipartite matchingM* between *X* and *Y* is a set of pairs *M* ⊆ *X* × *Y*, such that each element in *X* ∪ *Y* participates in at most one pair in *M*. If some element *z* ∈ *X* ∪ *Y* does not participate in any pair in *M*, we say that *z* is *unmatched* by *M* and denote *z* ∉ *M*. A (generalized) *matching cost functionw* for *X* and *Y* assigns costs *w*(*x*,*y*) for every (*x*,*y*) ∈ *X* × *Y* and costs *w*(*z*) for every *z* ∈ *X* ∪ *Y*. The *cost* of a bipartite matching *M* between *X* and *Y* with respect to *w* is given by

(1) $$w(M)=\underset{(x,y)\in M}{\sum }w(x,y)+\underset{z\in X\cup Y,\;z\notin M}{\sum }w(z)\;.$$

A *matching instance* is a triplet (*X*,*Y*,*w*), where *X* and *Y* are two sets, and *w* is a matching cost function for *X* and *Y*. The *Min-Cost Bipartite Matching* problem (*MCM*) is, given a matching instance (*X*,*Y*,*w*), to find the minimum cost of a matching between *X* and *Y* with respect to *w*. Denote by *MCM*(*X*,*Y*,*w*) the *solution* of the *MCM* problem for the instance (*X*,*Y*,*w*), and call a matching whose cost equals to the solution *optimal*.

Numerous works study and suggest algorithms for the *MCM* problem, usually when no unmatched element costs are taken into account (see e.g., [30-33]). A standard approach is to reduce *MCM* to the *Min-Cost Max-Flow* problem, which yields an *O*(*nm*^2^ + *m*^2^ log *m*) algorithm for *MCM*, where *n* = min(|*X*|,|*Y*|) and *m* = max(|*X*|,|*Y*|). In [27], an adapted reduction was presented which generalizes the problem definition to incorporate unmatched element costs and also runs in *O*(*nm*^2^ + *m*^2^ log *m*) time. An algorithm suggested by Dinitz in [29] solves the *MCM* problem in *O*(*n*^3^ + *nm*) time, without reducing it to Min-Cost Max-Flow. Since that paper is in Russian and since it considers neither unmatched element costs nor the cavity variants of *MCM* (see the following section), we prefer to explicitly adapt the Min-Cost Max-Flow approach to our needs, using some variation of the ideas of [29].

#### ***Cavity MCM***

The *All*-*Cavity*-*MCM* problem [28] is, given a matching instance (*X*,*Y*,*w*), to compute *MCM*(*X*,*Y* ∖ {*y*},*w*) for all *y* ∈ *Y*. We define the *All*-*Pairs*-*Cavity*-*MCM* problem as, given a matching instance (*X*,*Y*,*w*), to compute *MCM*(*X* ∖ {*x*},*Y* ∖ {*y*},*w*) for all *x* ∈ *X* and *y* ∈ *Y*.

Clearly, algorithms for both *All*-*Cavity*-*MCM* and *All*-*Pairs*-*Cavity*-*MCM* problems can be implemented by repeatedly running an algorithm for *MCM* on all required inputs. In [28], an algorithm for *All*-*Cavity*-*MCM* was proposed, which is more efficient than the naïve algorithm and retains the same cubic running time as the standard algorithm for *MCM*. To the best of our knowledge, no algorithm for *All*-*Pairs*-*Cavity*-*MCM* which improves upon the naïve algorithm (i.e. repeatedly executing the algorithm of [28] for *All*-*Cavity*-*MCM* over all *x* ∈ *X*) was previously described.

In Section ‘Algorithms for bipartite matching problems’, we give new algorithms for (generalized, unordered) *MCM*, *All*-*Cavity*-*MCM* and *All*-*Pairs*-*Cavity*-*MCM*. The running times of these algorithms are summarized in the following theorem, whose correctness is shown in Section ‘Algorithms for bipartite matching problems’.

#### 

**Theorem 1.** Let (*X*,*Y*,*w*) be a matching instance, and denote *n* = min(|*X*|,|*Y*|), *m* = max(|*X*|,|*Y*|). Then, each one of the problems *MCM*, *All*-*Cavity*-*MCM*, and *All*-*Pairs*-*Cavity*-*MCM* over the instance (*X*,*Y*,*w*) can be solved in *O*(*n*^3^ + *nm*) running time. Moreover, this may be done without the usage of priority queues (e.g. Fibonacci heaps [31]) or other complex data structures.

#### ***Ordered MCM variants***

For an ordered set *Z* = 〈 *z*_0_,*z*_1_,…,*z*_*n*-1_ 〉 and an integer *k*, the *k-rotation* of *Z* is the reordering of its elements $Z_{k}=\langle z_{0}^{\prime },z_{1}^{\prime },\ldots ,z_{n-1}^{\prime }\rangle$, where $z_{i}^{\prime }=z_{(i+k)\text{mod}n}$ (that is, *Z*_*k*_ = 〈 *z*_*k*_,*z*_*k* + 1_,…,*z*_*n*-1_,*z*_0_,…,*z*_*k*-1_ 〉). Note that *Z*_0_ = *Z*.

Let *X* and *Y* be two ordered sets, and *M* a bipartite matching between *X* and *Y*. Say that *Mpreserves linear order* if for every $\left(x_{i},y_{j}),(x_{i^{\prime }},y_{j^{\prime }}\right)$ ∈ *M*, *i* ≤ *i*′ ⇔ *j* ≤ *j*′. Say that *Mpreserves cyclic order* if there are some integers *k*,*l* such that *M* preserves linear order with respect to the rotated sets *X*_*k*_ and *Y*_*l*_. It is possible to show, that defining *M* as preserving cyclic order if there exists an integer *l* such that *M* preserves linear order with respect to *X* and *Y*_*l*_, is equivalent to the definition above.

The *Linear Ordered MCM* problem (*Linear-MCM*) and the *Cyclic Ordered MCM* problem (*Cyclic-MCM*) are defined similarly to *MCM*, with the restrictions that the considered matchings have to preserve linear or cyclic order, respectively. *Linear-MCM* is essentially equivalent to the Sequence Alignment problem, which can be solved in *O*(*nm*) running time [34]. *Cyclic-MCM* can be solved by taking the minimum cost solution among *Linear-MCM* solutions for all rotations of the smaller set in the input, in *O*(*n*^2^*m*). More efficient algorithms for *Cyclic-MCM* can be implied from [35-37].

### Homeomorphic subtree alignment

**Figure 2:**
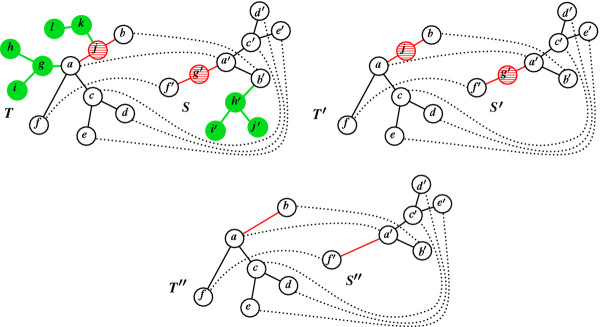
**Homeomorphic Subtree Alignment.** Thick lines represent tree edges, and dotted lines connect aligned node pairs. (**a**) An HSA *A* = {(*a*,*a*′),(*b*,*b*′),…,(*f*,*f*′)} between two trees *T* and *S*. The set of pruned subtrees (in green fillings) with respect to *A* is $\pi (A)=\left\{T_{g}^{a},T_{k}^{j},S_{h^{\prime }}^{b^{\prime }}\right\}$. The set of smoothed nodes (in lined red) with respect to *A* is *δ*(*A*) = {*j*,*g*′}. (**b**) The subtrees *T*′ and *S*′ of *T* and *S*, respectively, obtained after pruning the subtrees in *π*(*A*). (**c**) The smoothings *T*” and *S*” of *T*’ and *S*’, respectively, obtained after smoothing the nodes in *δ*(*A*). Mapping *A* is an isomorphic alignment between *T*” and *S*”.

An *isomorphic alignment* between two trees *T* = (*V*,*E*) and *S* = (*V*′,*E*′) is a bijection *A* : *V* → *V*′, such that for every pair of nodes *v*,*u* ∈ *V* we have that (*v*,*u*) ∈ *E* ⇔ (*A*(*v*),*A*(*u*)) ∈ *E* ′. A *homeomorphic alignment* between *T* and *S* is an isomorphic alignment between some smoothing *T*′ of *T* and some smoothing *S*′ of *S*, and a *homeomorphic subtree alignment* (HSA) between *T* and *S* is a homeomorphic alignment between some subtree *T*′ of *T* and some subtree *S*  ′ of *S* (Figure 2). For short, we write (*v*,*v*′) ∈ *A* to indicate that *A*(*v*) = *v*′.

Let *T* and *S* be two trees, and *A* a homeomorphic subtree alignment between them. Let *T*′ and *S*′ be the subtrees of *T* and *S*, and let *T*′′ and *S*′′ be the smoothings of *T*′ and *S*′, respectively, such that *A* is an isomorphic alignment between *T*′′ and *S*′′. Say that a node *v* ∈ *T* is *aligned* by *A* if *v* ∈ *T*′′, and that *v* is *smoothed* by *A* if *v* ∈ *T*′ and *v* ∉ *T*′′. Say that a subtree $T_{u}^{v}$ is *pruned* by *A* if *v* ∈ *T*′ and *u* ∉ *T*′. Let $\text{prune}(T_{u}^{v})$ be a cost associated with pruning the subtree $T_{u}^{v}$ from *T*, *smooth*(*v*) be a cost associated with smoothing node *v*, and *align*(*v*,*v*′) be a cost associated with aligning node *v* against some node *v*′. Definitions for *S* are similar. Denote by *π*(*A*) the set of pruned subtrees and by *δ*(*A*) the set of smoothed nodes of *T* and *S*, with respect to *A*. Define the *alignment cost*:

(2) $$\;w(T,S,A)\;=\;\underset{(v,v^{\prime })\in A}{\sum }\;\;\text{align}(v,v^{\prime })+\;\underset{T^{\prime }\in \pi (A)}{\sum }\;\text{prune}(T^{\prime })\;\underset{v\in \delta (A)}{\sum }\;\text{smooth}(v)\;.$$

Denote by *HSA*(*T*,*S*) the minimum alignment cost of an HSA between *T* and *S*, and call an HSA A *optimal* with respect to *T* and *S* if *w*(*T*,*S*,*A*) = *HSA*(*T*,*S*). The *Min-Cost HSA* problem is, given a pair of trees *T* and *S*, to compute *HSA*(*T*,*S*).

#### 

**Remark 1.** We do not give the details of how to construct optimal alignments in this paper. As usual for dynamic programming algorithms, this may be done by a standard back-tracking procedure applied on the computed dynamic programming tables.

#### ***Rooted and ordered alignments***

In addition to the general Min-Cost HSA problem, we also consider special cases of the problem in which the two input trees are rooted and/or ordered, and the alignment is required to satisfy certain restrictions with respect to these additional properties. For two rooted trees *T*^*v*^ and $S^{v\prime }$, say that *A* is a *rooted HSA* between *T*^*v*^ and $S^{v\prime }$ if *A* is an HSA between *T* and *S*, and (*v*,*v*′) ∈ *A*. The definition of *ordered HSA* requires some additional formalism, related to *bipartite matchings*.

Let *A* be an HSA between the trees *T* and *S*. For an edge (*v*→*u*) ∈ *T*, say that *u* is a *relevant neighbor* of *v* (with respect to *A*) if there is some $x\in T_{u}^{v},x\neq v$, which is aligned by *A*. Define relevant neighbors in *S* similarly.

#### 

**Observation 1.** Let *A* be an HSA between trees *T* and *S*, and (*v*,*v*′), (*x*,*x*′), (*y*,*y*′) ∈ *A*. The path between *x* and *y* in *T* goes through *v* if and only if the path between *x*′ and *y*′ in *S* goes through *v*′.

The correctness of the observation can be asserted from the fact that *A* is an isomorphic alignment between a smoothed subtree of *T* that contains *v*,*x*, and *y*, and a smoothed subtree of *S* that contains *v*′,*x*′, and *y*′.

#### 

**Lemma 1.** Let (*v*,*v*′) ∈ *A*, and let *u* be a relevant neighbor of *v*. Then, there is a unique relevant neighbor *u*′ of *v*′ such that for every (*y*,*y*′) ∈ *A*, $y\in T_{u}^{v}\Leftrightarrow y^{\prime }\in S_{u^{\prime }}^{v^{\prime }}$.

#### 

*Proof.* Since *u* is a relevant neighbor of *v*, there is a node $x\in T_{u}^{v}$, such that *x* ≠ *v* and *x* is aligned by *A*. Let *x*′ = *A*(*x*), and let *u*′ be the relevant neighbor of *v*′ such that $x^{\prime }\in S_{u^{\prime }}^{v^{\prime }}$. For (*y*,*y*′) ∈ *A*, observe that $y\notin T_{u}^{v}$ if and only if the path between *x* and *y* in *T* passes through *v*. Similarly, $y^{\prime }\notin S_{u^{\prime }}^{v^{\prime }}$ if and only if the path between *x*′ and *y*′ in *S* passes through *v*′. Applying Observation 1, this implies that $y\in T_{u}^{v}\Leftrightarrow y^{\prime }\in S_{u^{\prime }}^{v^{\prime }}$. □

**Figure 3:**
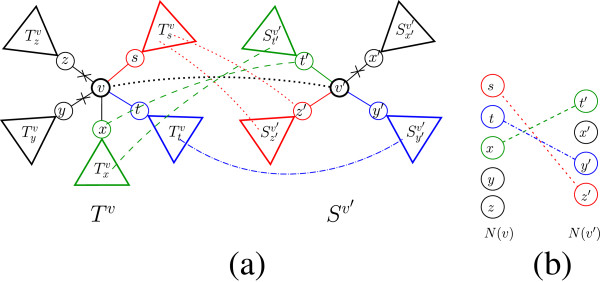
**An illustration of a rooted alignment*A *between*T***^***v ***^**and**$S^{v^{\prime }}$**.** (**a**) Each subtree of the form $T_{u}^{v}$ ($S_{u^{\prime }}^{v^{\prime }}$) is either pruned by the alignment, or matched to exactly one subtree of the form$S_{u^{\prime }}^{v^{\prime }}$ ($T_{u}^{v}$). In this example,$T_{s}^{v}$ is matched to$S_{z^{\prime }}^{v^{\prime }}$ (in red),$T_{t}^{v}$ is matched to$S_{y^{\prime }}^{v^{\prime }}$ (in blue), and$T_{x}^{v}$ is matched to$S_{t^{\prime }}^{v^{\prime }}$ (in green). These three subtree- matchings induce three sub-alignments of *A*:$A_{s}^{v}$,$A_{t}^{v}$, and$A_{x}^{v}$, respectively, where *A* is the union of these three sub-alignments (the pair (*v*,*v*′) participates in all three sub-alignments). The pruned subtrees in this example are $T_{y}^{v}$,$T_{z}^{v}$, and$S_{x^{\prime }}^{v^{\prime }}$. (**b**) The corresponding bipartite matching$M_{v,v^{\prime }}^{A}=\{(s,z^{\prime }),(t,y^{\prime }),(x,t^{\prime })\}$ between *N*(*v*) and *N*(*v*′).

Lemma 1 implies that for (*v*,*v*′) ∈ *A*, the alignment induces a bipartite matching $M_{v,v^{\prime }}^{A}$ between *N*(*v*) and *N*(*v*′) in which the matched elements are exactly those relevant neighbors of *v* and *v*′ (Figure 3). Say that *A* is *ordered* if *T* and *S* are ordered trees, and for every (*v*,*v*′)∈*A*, the corresponding bipartite matching $M_{v,v^{\prime }}^{A}$ is cyclically ordered.

Now, we can define three additional variants of the HSA problem. Let *T*^*v*^ and$S^{v^{\prime }}$ be rooted and ordered trees. Denote by *Ordered-HSA*(*T*,*S*),$\text{Rooted-HSA}(T^{v},S^{v^{\prime }})$ and$\text{Ordered-Rooted-HSA}(T^{v},S^{v^{\prime }})$ the minimum costs of an ordered HSA, a rooted HSA, and an ordered and rooted HSA between *T*^*v*^ and $S^{v^{\prime }}$, respectively. Define the corresponding variants of the Min-Cost HSA problem whose goals are to compute these values.

## Algorithm for homeomorphic subtree alignment

In this section we describe a basic algorithm for HSA for its unordered unrooted variant (though it is adequate for the other variants as well with some simple modifications).

### Recursive computation

Let *A* be an HSA between *T* and *S*. Let (*v*,*v*′) ∈ *A*, and $M_{v,v^{\prime }}^{A}$ the corresponding bipartite matching between *N*(*v*) and *N*(*v*′), as defined in Section ‘Homeomorphic subtree alignment’. Note that *A* can be viewed as a rooted alignment between *T*^*v*^ and $S^{v^{\prime }}$, which is the union of a set of rooted sub-alignments$A_{u}^{v}$ between rooted subtree pairs of the form $T_{u}^{v}$ and $S_{u^{\prime }}^{v^{\prime }}$, where$(u,u^{\prime })\in M_{v,v^{\prime }}^{A}$ (Figure 3). The alignment cost can therefore be obtained by summing the costs of these sub-alignments, which cover all scoring terms implied by matching nodes, smoothing nodes, and pruning subtrees by the corresponding sub-alignments, and the additional pruning costs of pruned subtrees of the forms$T_{u}^{v}$ and$S_{u^{\prime }}^{v^{\prime }}$ (where *u*,*u*′ are unmatched by $M_{v,v^{\prime }}^{A}$). Note that the pair (*v*,*v*′) belongs by definition to each of the sub-alignments $A_{u}^{v}$. In order to avoid multiple additions of the term *align*(*v*,*v*′) when summing sub-alignment costs, define $w^{-r}(T^{v},S^{v^{\prime }},A)=w(T^{v},S^{v^{\prime }},A)-\text{align}(v,v^{\prime })$. The cost $w(T^{v},S^{v^{\prime }},A)$ can then be written as follows:

(3) $$\begin{array}{lll} \; & w(T^{v},S^{v^{\prime }},A)=w^{-r}(T^{v},S^{v^{\prime }},A)+\text{align}(v,v^{\prime }),\;\\ \; & w^{-r}(T^{v},S^{v^{\prime }},A)=\;\underset{\begin{array}{c} u\in N(v),\\ u\notin M_{v,v^{\prime }}^{A} \end{array}}{\sum }\;\text{prune}(T_{u}^{v})+\;\underset{\begin{array}{c} u^{\prime }\in N(v^{\prime }),\\ u^{\prime }\notin M_{v,v^{\prime }}^{A} \end{array}}{\sum }\;\text{prune}(S_{u^{\prime }}^{v^{\prime }})\; & \end{array}$$

(4) $$\begin{array}{ll} & \;+\underset{(u,u^{\prime })\in M_{v,v^{\prime }}^{A}}{\sum }w^{\text{-r}}(T_{u}^{v},S_{u^{\prime }}^{v^{\prime }},A_{u}^{v})\;.\; \end{array}$$

Call a rooted alignment *non-trivial* if it aligns at least one additional pair of nodes besides the roots. Note that every rooted sub-alignment$A_{u}^{v}$ is non-trivial (since *u* and *u*′ are relevant neighbors of *v* and *v*′). Denote by ${\text{Rooted-HSA}}^{-r}\left(T_{u}^{v},S_{u^{\prime }}^{v^{\prime }}\right)$ the minimum *w*^-*r*^ cost of a non-trivial rooted alignment between $T_{u}^{v}$ and $S_{u^{\prime }}^{v^{\prime }}$.

Clearly, if *A* is an optimal rooted HSA between *T*^*v*^ and $S^{v^{\prime }}$, then for each$(u,u^{\prime })\in M_{v,v^{\prime }}^{A}$$w^{-r}\left(T_{u}^{v},S_{u^{\prime }}^{v^{\prime }},A_{u}^{v}\right)={\text{Rooted-HSA}}^{-r}\left(T_{u}^{v},S_{u^{\prime }}^{v^{\prime }}\right)$ (otherwise, it is possible to produce a rooted alignment with a better cost than *A* for *T*^*v*^ and $S^{v^{\prime }}$). Define the bipartite matching instance $\left(N(v),N(v^{\prime }),w_{v,v^{\prime }}\right)$, where for *u*∈*N*(*v*), *u*′∈*N*(*v*′), se$w_{v,v^{\prime }}(u,u^{\prime })={\text{Rooted-HSA}}^{-r}(T_{u}^{v},S_{u^{\prime }}^{v^{\prime }}),\;w_{v,v^{\prime }}(u)=\text{prune}(T_{u}^{v})$, and $w_{v,v^{\prime }}(u^{\prime })=\text{prune}(S_{u^{\prime }}^{v^{\prime }})$. Observe that the right-hand side of Equation 4 equals the cost of the bipartite matching$M_{v,v^{\prime }}^{A}$ for the matching instance$\left(N(v),N(v^{\prime }),w_{v,v^{\prime }}\right)$ (see Figure 3b). In addition, every bipartite matching between *N*(*v*) and *N*(*v*′) corresponds to some valid rooted HSA between *T*^*v*^ and $S^{v\prime }$, so that the matching and alignment costs are equal.

Therefore, a minimum cost bipartite matching induces a minimum cost alignment, and we get that

(5) $$\;\text{Rooted-HSA}(T^{v},S^{v^{\prime }})\;=\;\text{align}(v,v^{\prime })+\text{MCM}\left(N(v),N(v^{\prime }),w_{v,v^{\prime }}\right)\;.$$

Assuming the non-degenerate case where an optimal HSA between *T* and *S* contains at least one pair (*v*,*v*′), we can compute the cost of an optimal HSA by solving *Rooted-HSA* with respect to all possible root pairs, and taking the pair which induces a minimum cost as a solution for the unrooted case:

(6) $$\text{HSA}(T,S)=\underset{v\in T,v^{\prime }\in S}{\text{min}}\text{Rooted-HSA}(T^{v},S^{v^{\prime }})\;.$$

**Figure 4:**
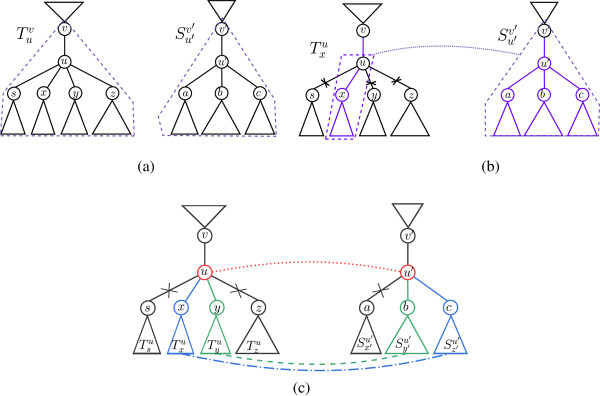
**An illustration of the computation of Equation**7**.** (**a**) The instance$\left(T_{u}^{v},S_{u^{\prime }}^{v^{\prime }}\right)$ in the left-hand side of the equation. (**b**) The computation of term I in the right-hand side of the equation: The term considers the best alignment score under the assumption that *u* is smoothed. In this case, the score is obtained by taking the smoothing cost of *u*, and summing it, for some *x* ∈ *N*(*u*) ∖ {*v*}, with the alignment score between$T_{x}^{u}$ and$S_{u^{\prime }}^{v^{\prime }}$ and the pruning cost of all subtrees$T_{y}^{u}$ for *y* ∈ *N*(*u*)∖{*v*,*x*}. Vertex *x* is chosen to be the neighbor of *u* that induces a minimum cost with respect to this computation. The computation of term II is symmetric. (**c**) The computation of term III in the right-hand side of the equation: This term considers the case were neither *u* nor *u*′ are smoothed, and therefore these two nodes are aligned to each other. In this case, the score is computed similarly to the computation in Equation 5, with respect to the sets *N*(*u*) ∖ {*v*} and *N*(*u*′)∖{*v*′**}.**

In order to obtain cost functions of the form$w_{v,v^{\prime }}$ for the computation of Equation 5, we need to compute solutions of the form${\text{Rooted-HSA}}^{-r}(T_{u}^{v},S_{u^{\prime }}^{v^{\prime }})$ for sub-instances of the input. When *u* and *u*′ are leaves, the only non-trivial rooted alignment between $T_{u}^{v}$ and$S_{u^{\prime }}^{v^{\prime }}$ contains both pairs (*v*,*v*′) and (*u*,*u*′), and therefore we get that${\text{Rooted-HSA}}^{-r}\left(T_{u}^{v},S_{u^{\prime }}^{v^{\prime }}\right)=\text{align}(u,u^{\prime })$. Otherwise, Equation 7 computes${\text{Rooted-HSA}}^{-r}\left(T_{u}^{v},S_{u^{\prime }}^{v^{\prime }}\right)$ recursively (see Figure 4), where$w_{u,u^{\prime }}^{v,v^{\prime }}$ is defined similarly to$w_{u,u^{\prime }}$ with respect to the sets *N*(*u*) ∖ {*v*} and *N*(*u*′)∖{*v*′}.

(7) $$\begin{array}{c} {\text{Rooted-HSA}}^{-r}\left(T_{u}^{v},S_{u^{\prime }}^{v^{\prime }}\right)=\\ \text{min}\left\{\begin{array}{c} I.\text{smooth}(u)+\underset{x\in N(u)\setminus \{v\}}{\text{min}}\left({\text{Rooted-HSA}}^{-r}\left(\overset{u}{\underset{x}{T}},\overset{v^{\prime }}{\underset{u^{\prime }}{S}}\right)+\underset{y\in N(u)\setminus \{v,x\}}{\sum }\text{prune}(\overset{u}{\underset{y}{T}})\right),\\ \text{II}.\text{smooth}(u^{\prime })+\underset{x^{\prime }\in N(u^{\prime })\setminus \{v^{\prime }\}}{\text{min}}\left({\text{Rooted-HSA}}^{-r}\left(\overset{v}{\underset{u}{T}},\overset{u^{\prime }}{\underset{x^{\prime }}{S}}\right)+\underset{y^{\prime }\in N(u^{\prime })\setminus \{v^{\prime },x^{\prime }\}}{\sum }\text{prune}(\overset{u^{\prime }}{\underset{y^{\prime }}{S}})\right),\\ \text{III}.\text{align}(u,u^{\prime })+\text{MCM}\left(N(u)\setminus \{v\},N(u^{\prime })\setminus \{v^{\prime }\},w_{u,u^{\prime }}^{v,v^{\prime }}\right) \end{array}\right\} \end{array}$$

#### 

*Proof.* [Equation 7]. By definition of the *Rooted-HSA*^-*r*^$\left(T_{u}^{v},S_{u^{\prime }}^{v^{\prime }}\right)$ score, it corresponds to the score of the best non-trivial alignment between$T_{u}^{v}$ and$S_{u^{\prime }}^{v^{\prime }}$, minus the root alignment cost term *align*(*v*,*v*′). We may cover the set of all possible non-trivial rooted alignments between$T_{u}^{v}$ and$S_{u^{\prime }}^{v^{\prime }}$ by three sets: (a) alignments in which *u* is unmatched, (b) alignments in which *u*′ is unmatched, and (c) alignments in which both *u* and *u*′ are matched (note that there might be an intersection between groups (*a*) and (*b*)). We show that each one of the terms I, II, and III in the right-hand side of Equation 7 computes the minimum cost of an alignment in each one of the groups (a), (b), and (c), respectively, and therefore the minimum among all these terms gives the correct value${\text{Rooted-HSA}}^{-r}\left(T_{u}^{v},S_{u^{\prime }}^{v^{\prime }}\right)$.

We start by showing that term I computes the minimum cost of an alignment in group (a). Let *A* be an alignment in group (a) of minimum cost. Since *A* is non-trivial, and *u* is smoothed in *A*, *u* has exactly one additional relevant neighbor *x*^∗^ besides *v*. It therefore follows that all nodes in$T_{u}^{v}$ except for *v* which are matched by *A* belong to the subtree$T_{x^{*}}^{u}$. Defining$A_{x^{*}}^{u}=\left(A\setminus \{(v,v^{\prime })\}\right)\cup \{(u,v^{\prime })\}$, we have that$A_{x^{*}}^{u}$ is a non-trivial rooted HSA between$T_{x^{*}}^{u}$ and$S_{u^{\prime }}^{v^{\prime }}$. Therefore, the cost of *A* is obtained by the summation of pruning costs of all subtrees$T_{y}^{u}$ for *y* ∈ *N*(*u*) ∖ {*v*,*x*^∗^}, the cost of smoothing *u*, and the cost$w^{-r}\left(T_{x^{*}}^{u},S_{u^{\prime }}^{v^{\prime }},A_{x^{*}}^{u}\right)$ (which counts for all cost terms corresponding to node matchings, node smoothings, and subtree prunings, implied by the sub-alignment$A_{x^{*}}^{u}$). Since *A* is optimal, it is clear that$w^{-r}\left(T_{x^{*}}^{u},S_{u^{\prime }}^{v^{\prime }},A_{x^{*}}^{u}\right)={\text{Rooted-HSA}}^{-r}\left(T_{x^{*}}^{u},S_{u^{\prime }}^{v^{\prime }}\right)$ (otherwise, it is possible to improve the cost of *A* by replacing the sub-alignment$A_{x^{*}}^{u}$ with an optimal alignment for the corresponding sub-instance). Thus,

$$\begin{array}{c} w^{\text{-r}}\left(T_{u}^{v},S_{u^{\prime }}^{v^{\prime }},A\right)=\text{smooth}(u)+{\text{Rooted-HSA}}^{-r}\left(T_{x^{*}}^{u},S_{u^{\prime }}^{v^{\prime }}\right)\\ \;+\underset{y\in N(u)\setminus \{v,x^{*}\}}{\sum }\text{prune}(T_{y}^{u})\;. \end{array}$$

Since for every *x* ∈ *N*(*u*) ∖ {*v*} the term

$$\text{smooth}(u)+\text{Rooted}-\text{HS}A^{-r}\left(T_{x}^{u},S_{u^{\prime }}^{v^{\prime }}\right)+\;\;\underset{y\in N(u)\setminus \{v,x\}}{\sum }\;\text{prune}(T_{y}^{u})$$

is the *w*^-r^ cost of a possible non-trivial alignment between$T_{u}^{v}$ and$S_{u^{\prime }}^{v^{\prime }}$ in which *u* is smoothed (where the subtree$T_{x}^{u}$ is optimally aligned to$S_{u^{\prime }}^{v^{\prime }}$), we get that *x*^∗^ satisfies that

$$\begin{array}{lll} \; & \text{smooth}(u)+\;\underset{y\in N(u)\setminus \{v,x^{*}\}}{\sum }\;\text{prune}(T_{y}^{u})\;+\;\text{Rooted}\;-\;\text{HS}A^{-r}\;\left(\;T_{x^{*}}^{u},S_{u^{\prime }}^{v^{\prime }}\;\right)\;=\; & \\ \; & \text{smooth}(u)\;+\;\underset{x\in N(u)\setminus \{v\}}{\text{min}}\;\left(\underset{y\in N(u)\setminus \{v,x\}}{\sum }\;\text{prune}(T_{y}^{u})\;+\;\text{Rooted}\;-\;\text{HSA}{\;}^{-r}\;\left(\;T_{x}^{u}\;,\;S_{u^{\prime }}^{v^{\prime }}\;\right)\right),\; & \end{array}$$

hence the correctness of term I.

The proof that term **II** of the equation computes the minimum cost of an alignment in group (b) is symmetric to the proof of term **I**. As for term **III**, note that the case where *u* and *u*′ are matched, but not to each other, implies a contradiction to Observation 1. Therefore, for any alignment in group (c), and in particular for such an alignment *A* of minimum cost, we have that (*u*,*u*′) ∈ *A*. In this case, the optimal alignment cost is obtained immediately from applying Equation 5 for this specific sub-instance, as formulated by term **III** in the equation. □

The computation of$w_{u,u^{\prime }}^{v,v^{\prime }}$ requires the computation of scores of the form${\text{Rooted-HSA}}^{-r}\left(T_{x}^{u},S_{x^{\prime }}^{u^{\prime }}\right)$ for all *x* ∈ *N*(*u*) ∖ {*v*} and all *x*′ ∈ *N*(*u*′) ∖ {*v*′}. It can be shown that all *Rooted-HSA*^-*r*^ solutions required for the computation of the right-hand side of the equation are for strictly smaller sub-instances than the sub-instance appearing in the left-hand side, thus the termination of the recursive computation is guaranteed. Equation 7 can be efficiently computed using Dynamic Programming (DP), as summarized by Algorithm 1 below.

#### **Algorithm 1:** *HSA* (*T*,*S*)

In Section ‘Time complexity of Algorithm 1’, we show that a straightforward implementation of Algorithm 1 obtains the running time of *O*(min(*d*_*T*_,*d*_*S*_)*n*_*T*_*n*_*S*_ + min (*d*_*T*_,*d*_*S*_)^3^*L*_*T*_*L*_*S*_). For some trees *T*,*S* with *n*_*T*_,*L*_*T*_,*d*_*T*_, *n*_*S*_,*L*_*S*_,*d*_*S*_ = *Θ*(*n*) (e.g. “star” trees), this implies an *O*(*n*^5^) running time. In Section ‘Improving the time complexity’, we show how to improve this time bound and obtain a cubic time algorithm for the problem.

We note that Algorithm 1 generalizes to also solve the ordered unrooted, unordered rooted, and ordered rooted variants of *Min-Cost HSA* in polynomial time. In case a rooted alignment is sought, the algorithm can compute Equation 5 in line 3 only with respect to the two roots, and avoid the computation of Equation 6. In case an ordered alignment is sought, the *MCM* application in Equation 5 can be replaced by *Cyclic-MCM*, and in Equation 7*MCM* can be replaced by *Linear-MCM*, similarly to [27]. Traditionally, ordered matchings are implemented via reduction to sequence alignment [7,26,38]. Similarly to the improvement described in the next section for the unordered tree alignment algorithm, it seems that fast incremental/decremental versions of ordered matchings [35-37] can be integrated into the ordered variant of our algorithm to improve its time complexity. However, the detailed description of these techniques are beyond the scope of this paper.

### Time complexity of Algorithm 1

We first analyze the time complexity of a straightforward implementation of the algorithm. Then, in Section ‘Improving the time complexity’, we show how this time complexity can be reduced by applying *cavity matching* subroutines.

Let$\text{index}(T_{u}^{v})$ and$\text{index}(S_{u^{\prime }}^{v^{\prime }})$ denote the row and column indices of$T_{u}^{v}$ and$S_{u^{\prime }}^{v^{\prime }}$ in *H*, respectively. Let *u* ∈ *T* and *u*′ ∈ *S* be a pair of nodes. The set of subtrees$T_{u}^{v}$ for *v* ∈ *N*(*u*) corresponds to a subset of rows in *H*. Similarly, the set of subtrees$S_{u^{\prime }}^{v^{\prime }}$ for *v*′ ∈ *N*(*u*′) corresponds to a subset of columns in *H*, and thus all solutions of the form${\text{Rooted-HSA}}^{-r}\left(T_{u}^{v},S_{u^{\prime }}^{v^{\prime }}\right)$ are stored in a sub-matrix of *H* of size$d_{u}\times d_{u^{\prime }}$. Let$H_{u,u^{\prime }}$ denote this sub-matrix, and let *v*_*i*_ and$v_{j}^{\prime }$ denote nodes in *N*(*u*) and *N*(*u*′) such that$T_{u}^{v_{i}}$ and$S_{u^{\prime }}^{v_{j}^{\prime }}$ correspond to the *i*-th row and *j*-th column in$H_{u,u^{\prime }}$, respectively (i.e.$\text{index}(T_{u}^{v_{1}})$ is the first row in$H_{u,u^{\prime }}$,$\text{index}(T_{u}^{v_{2}})$ is the second row, etc.). Note that *H* can be viewed as a union of sub-matrices of the form$H_{u,u^{\prime }}$, where each entry in *H* is covered by exactly one sub-matrix$H_{u,u^{\prime }}$ for some *u* ∈ *T*, *u*′ ∈ *S*.

**Figure 5:**
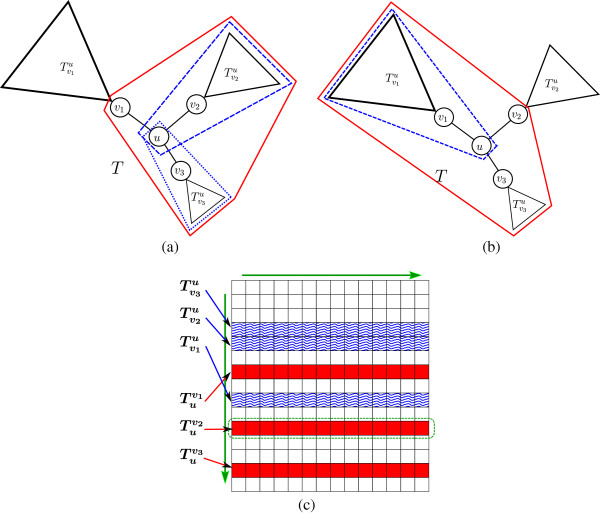
**An illustration of Observation**2**.** (**a**) A node *u* in a tree *T*, such that *N*(*u*) = {*v*_**1**_,*v*_**2**_,*v*_**3**_}, and$\left|T_{u}^{v_{1}}|\leq |T_{u}^{v_{2}}|\leq |T_{u}^{v_{3}}\right|$. The subtree$T_{u}^{v_{1}}$ (bounded by a solid red line) contains$T_{v_{2}}^{u}$ and$T_{v_{3}}^{u}$ as subtrees (bounded by dashed and dotted blue lines, respectively), and therefore$\left|T_{v_{2}}^{u}|,|T_{v_{3}}^{u}|\leq |T_{u}^{v_{1}}|\leq |T_{u}^{v_{2}}\right|$. (**b**) The subtree$T_{u}^{v_{2}}$ (bounded by a solid red line) contains$T_{v_{1}}^{u}$ as a subtree (bounded by a dashed blue line), and therefore$\left|T_{v_{1}}^{u}|,|T_{v_{2}}^{u}|,|T_{v_{3}}^{u}|\leq |T_{u}^{v_{2}}\right|$. (**c**) The DP matrix *H*. The rows of the matrix correspond to subtrees of *T*, sorted from top to bottom with non-decreasing tree size. Rows corresponding to subtrees of the form$T_{u}^{v_{i}}$ are in solid red, and rows corresponding to subtrees of the form$T_{v_{i}}^{u}$ are in waved-blue. All waved-blue rows have smaller indices than the row corresponding to$T_{u}^{v_{2}}$ (circled with a dashed green line).

The following observation identifies special properties of the second column and second row in$H_{u,u^{\prime }}$, which are exploited for the efficient computation of Algorithm 1. Observe that for every 1 < *i* ≤ *d*_*u*_,$T_{v_{i}}^{u}$ is a subtree of$T_{u}^{v_{1}}$, and therefore$\text{index}(T_{v_{i}}^{u})<\text{index}(T_{u}^{v_{1}})$. Also,$T_{v_{1}}^{u}$ is a subtree of$T_{u}^{v_{2}}$, and therefore$\text{index}(T_{v_{1}}^{u})<\text{index}(T_{u}^{v_{2}})$. Since$\text{index}(T_{u}^{v_{1}})<\text{index}(T_{u}^{v_{2}})$, we get the following observation (Figure 5):

#### 

**Observation 2.** For every 1 ≤ *i* ≤ *d*_*u*_,$\text{index}(T_{v_{i}}^{u})<\text{index}(T_{u}^{v_{2}})$. Similarly, for every$1\leq j\leq d_{u^{\prime }}$,$\text{index}(S_{v_{j}^{\prime }}^{u})<\text{index}(S_{u^{\prime }}^{v_{2}^{\prime }})$.

In order to focus on the bottleneck expression in the running time analysis of the algorithm, we first summarize the complexity of its secondary computations in the following lemma:

#### 

**Lemma 2.** It is possible to implement Algorithm 1 so that all operations, besides computation of solutions to the *MCM* problem, require *O*(*n*_*T*_*n*_*S*_) running time.

#### 

*Proof.* It is simple to observe that the computations conducted in lines 1 and 4 of the algorithm consume *O*(*n*_*T*_*n*_*S*_) time (the computation of all subtree sizes and their sorting can be implemented in a linear time in a straightforward manner, where the details are omitted from this text). As computations of Equation 5 in line 3 and of term **III** of Equation 7 in line 2 are dominated by *MCM* computations, it remains to show that it is possible to compute terms I and II of Equation 7 in line 2 of the algorithm in *O*(*n*_*T*_*n*_*S*_) along the entire run of the algorithm.

**Figure 6:**
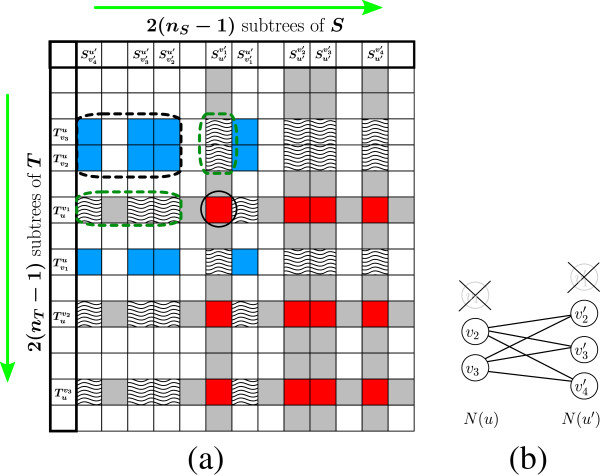
**The incorporation of cavity matching subroutines in the DP algorithm.** In (a), the DP matrix *H* is illustrated, where the solid red entries correspond to entries in the sub-matrix$H_{u,u^{\prime }}$ for some *u* ∈ *T* and *u*′∈*S*. In this example, *N*(*u*) = *v*_**1**_,*v*_**2**_,*v*_**3**_, and$N(u^{\prime })=v_{1}^{\prime },v_{2}^{\prime },v_{3}^{\prime },v_{4}^{\prime }$, where$\left|T_{u}^{v_{1}}|\leq |T_{u}^{v_{2}}|\leq |T_{u}^{v_{3}}\right|$, and$\left|S_{u^{\prime }}^{v_{1}^{\prime }}|\leq |S_{u^{\prime }}^{v_{2}^{\prime }}|\leq |S_{u^{\prime }}^{v_{3}^{\prime }}|\leq |S_{u^{\prime }}^{v_{4}^{\prime }}\right|$. The solid blue entries correspond to computed values of the form${\text{Rooted-HSA}}^{-r}(T_{v_{i}}^{u},S_{v_{j}^{\prime }}^{u^{\prime }})$, which are required for the computation of term **III** in Equation 7 for entries in$H_{u,u^{\prime }}$. The waived entries correspond to computed values of the form${\text{Rooted-HSA}}^{-r}(T_{v_{i}}^{u},S_{u^{\prime }}^{v_{j}^{\prime }})$ and${\text{Rooted-HSA}}^{-r}(T_{u}^{v_{i}},S_{v_{j}^{\prime }}^{u^{\prime }})$, which are required for the computation of terms **I** and **II** in Equation 7 for entries in$H_{u,u^{\prime }}$. (**a**) The computation of${\text{Rooted-HSA}}^{-r}(T_{u}^{v_{1}},S_{u^{\prime }}^{v_{1}^{\prime }})$ according to Equation 7. The entry corresponding to this sub-instance is marked with a solid black circle. In order to compute term **I** of the equation, there is a need to examine values of the form${\text{Rooted-HSA}}^{-r}(T_{v_{j}}^{u},S_{u^{\prime }}^{v_{1}^{\prime }})$ for *v*_*j*_ ∈ *N*(*u*) ∖ {*v*_**1**_}. As each$T_{v_{j}}^{u}$ is a subtree of$T_{u}^{v_{1}}$, the rows corresponding to these subtrees have smaller indices than the row of$T_{u}^{v_{1}}$, and so the required solutions are already computed and stored in *H* (waved entries marked with a dashed green circle, in the same column above the computed entry). Similarly, for computing term **II**, there is a need to examine solutions${\text{Rooted-HSA}}^{-r}(T_{u}^{v_{1}},S_{v_{j}^{\prime }}^{u^{\prime }})$ for$v_{j}^{\prime }\in N(u^{\prime })\setminus \{v_{1}^{\prime }\}$, appearing at the same row and to the left of the computed entry. For computing term **III**, there is a need to construct the matching cost function$w_{u,u^{\prime }}^{v_{1},v_{1}^{\prime }}$, which assigns for each *v*_*j*_ ∈ *N*(*u*) ∖ {*v*_**1**_} and$v_{j^{\prime }}^{\prime }\in N(u^{\prime })\setminus \{v_{1}^{\prime }\}$ the matching cost$w_{u,u^{\prime }}^{v_{1},v_{1}^{\prime }}(v_{j},v_{j^{\prime }}^{\prime })={\text{Rooted-HSA}}^{-r}(T_{v_{j}}^{u},S_{v_{j^{\prime }}^{\prime }}^{u^{\prime }})$ (the corresponding matching instance is shown in (**b**)). These required values appear in blue entries marked by a dashed black circle. Due to the order in which entries are being traversed, all required values were previously computed by the algorithm and stored in *H*, thus it is possible to compute${\text{Rooted-HSA}}^{-r}(T_{u}^{v_{1}},S_{u^{\prime }}^{v_{1}^{\prime }})$ at this stage.

Consider a pair of nodes *u* ∈ *T* and *u*′∈*S*, and the corresponding sub-matrix$H_{u,u^{\prime }}$ (for illustration, here and on, see Figure 6a). It is simple to observe that an explicit computation of term I of the equation with respect to some subtrees$T_{u}^{v}$ and$S_{u^{\prime }}^{v^{\prime }}$ can be conducted in *O*(*d*_*u*_) time. Nevertheless, we next show how to conduct this computation in *O*(1) amortized time. Fix an index$1\leq j\leq d_{u^{\prime }}$. Let$x^{*}={\text{argmin}}_{x\in N(u)}\left({\text{Rooted-HSA}}^{-r}\left(T_{x}^{u},S_{u^{\prime }}^{v_{j}^{\prime }}\right)-\text{prune}(T_{x}^{u})\right)$, and denote$\alpha ={\text{Rooted-HSA}}^{-r}\left(T_{x^{*}}^{u},S_{u^{\prime }}^{v_{j}^{\prime }}\right)-\text{prune}(T_{x^{*}}^{u})$. As *N*(*u*) ∖ {*v*} ⊂ *N*(*u*) for every *v* ∈ *N*(*u*), it is clear that for *v* ≠ *x*^∗^,min*x* ∈ *N*(*u*) ∖ {*v*}$\left({\text{Rooted-HSA}}^{-r}\left(T_{x}^{u},S_{u^{\prime }}^{v_{j}^{\prime }}\right)-\text{prune}(T_{x}^{u})\right)=\alpha \;$. Similarly, denote$\beta =\underset{x\in N(u)}{\sum }\text{prune}(T_{x}^{u})$, and observe that$\underset{x\in N(u)\setminus \{v\}}{\sum }\text{prune}(T_{x}^{u})=\beta -\text{prune}(T_{v}^{u})$ for every *v* ∈ *N*(*u*). Thus, for *v* ≠ *x*^∗^, term I of Equation 7 can be written as$\text{smooth}(u)+\beta -\text{prune}(T_{v}^{u})+\alpha$, and given the values *α* and *β*, be computed in *O*(1) time. Due to Observation 2, when the algorithm is about to compute the entry in the second row and *j*-th column of$H_{u,u^{\prime }}$ (i.e. the entry corresponding to$T_{u}^{v_{2}}$ and$S_{u^{\prime }}^{v_{j}^{\prime }}$), all required values for computing *x*^∗^,*α*, and *β*, are already stored in *H*, and therefore these values may be computed in *O*(*d*_*u*_) time. Once computing these values, it is possible to compute term I for each of the remaining rows *i* > 1 in column *j* of$H_{u,u^{\prime }}$, except for row *i* such that *v*_*i*_ = *x*^∗^, in *O*(1) time each. Additional *O*(*d*_*u*_) operations are required for computing the term for row 1 and the row *i* such that *v*_*i*_ = *x*^∗^, and therefore the total number of operations for computing the term for all *d*_*u*_ entries in column *j* of$H_{u,u^{\prime }}$ is *O*(*d*_*u*_). This implies that the amortized time for computing term **I** for each entry in$H_{u,u^{\prime }}$ is *O*(1), and therefore the amortized time for computing term I for each entry in *H* is *O*(1). The proof for term II is symmetric. All in all, we get that the running time for all operations conducted by the algorithm, besides *MCM*vcomputations, is *O*(*n*_*T*_*n*_*S*_). □

Before continuing with the time complexity analysis, we formulate an auxiliary lemma.

#### 

**Lemma 3.** For a tree *T*,$\underset{v\in T}{\sum }d_{v}=O(n_{T})$, and∑v∈T,dv≥3dv=O(LT).

#### 

*Proof.* It is well known that the number of undirected edges in *T* is *n*_*T*_**-1. Since each undirected edge (***v*,*u*) contributes 1 unit to the degrees *d*_*v*_ and *d*_*u*_ of its endpoints, we get that$\underset{v\in T}{\sum }d_{v}=2(n_{T}-1)=O(n_{T})$.In order to show that∑v∈T,dv≥3dv=O(LT), we will show that∑v∈Tdv≥3dv<3LT. When *T* contains a single node, *L*_*T*_ = 1,∑v∈Tdv≥3dv=0,and the inequality follows. Assume by induction the correctness of the inequality for all trees with less than *n* > 1 nodes, and let *T* be a tree with *n* nodes. Let (*x*,*y*) ∈ *T* be an edge such that *y* is leaf in *T*. The subtree *T*′ of *T* containing all nodes in *T* except for *y* (and all edges except for (*x*,*y*)) is of size *n* - 1, and from the inductive assumption∑v∈T′dv′≥3dv′<3LT′, where$d_{v}^{\prime }$ denotes the degree of *v* in *T*′. Besides *y*, *T* contains no additional leaves which are not already leaves in *T*′. In addition,$d_{v}=d_{v}^{\prime }$ for all nodes *v* ∈ *T*′ besides *x*, where$d_{x}=d_{x}^{\prime }+1$, and *d*_*y*_ = 1. If *x* is a leaf in *T*′ then$L_{T}=L_{T^{\prime }}$ (as *y* replaces *x* as a leaf in *T*), and since$d_{x}=d_{x}^{\prime }+1<3$ we get that∑v∈Tdv≥3dv=∑v∈T′dv′≥3dv′<3LT′=3LT. If *x* is not a leaf in *T*′ then$L_{T}=L_{T^{\prime }}+1$ (due to the addition of *y* as a leaf). Here, it is possible that$d_{x}^{\prime }=2$ and *d*_*x*_ = 3, which would contribute 3 to the degree summation for nodes with degree ≥ 3, while if$d_{x}^{\prime }>2$ the increment in the degree of *x* would contribute 1 to this summation. Therefore,

∑v∈Tdv≥3dv≤3+∑v∈T′dv′≥3dv′<3+3LT′=3LT,

and the lemma follows. □

In order to complete the time complexity analysis, we turn to count the number of operations applied in *MCM* computations throughout the algorithm’s run. Such computations are applied when computing term **III** of Equation 7, or when computing Equation 5. Term III of Equation 7 is computed once for every pair of subtrees$T_{u}^{v}$ and$S_{u^{\prime }}^{v^{\prime }}$, in$O(\text{min}{\left(d_{u},d_{u^{\prime }}\right)}^{3}+d_{u}d_{u^{\prime }})$ running time (Theorem 1, see also Section ‘Reducing *MCM* to *Min-Cost Max-Flow*’). Therefore, for a given pair of nodes *u* ∈ *T* and *u*′ ∈ *S*, and all neighbor pairs *v* ∈ *N*(*u*),*v*′ ∈ *N*(*u*′), the time required for *MCM* computations due to term III is

∑v∈N(u),v′∈N(u′)min(du,du′)3+dudu′=dudu′min(du,du′)3+du2du′2.

In order to sum the expression above for all pairs *u* ∈ *T*, *u*′ ∈ *S*, we sum indempendetly the expressions$\underset{u\in T}{\sum }\underset{u^{\prime }\in S}{\sum }d_{u}d_{u^{\prime }}\text{min}{\left(d_{u},d_{u^{\prime }}\right)}^{3}$ and$\underset{u\in T}{\sum }\underset{u^{\prime }\in S}{\sum }d_{u}^{2}d_{u^{\prime }}^{2}$:

∑u∈T∑u′∈Sdudu′min(du,du′)3≤∑u∈Tdu<3∑u′∈S8dudu′+∑u∈T∑u′∈Sdu′<38dudu′+∑u∈Tdu≥3∑u′∈Sdu′≥3dudu′min(dT,dS)3≤16∑u∈Tdu∑u′∈Sdu′+min(dT,dS)3∑u∈Tdu≥3du∑u′∈Sdu′≥3du′=Lem.3O(nTnS+min(dT,dS)3LTLS),∑u∈T∑u′∈Sdu2,du′2≤∑u∈Tdu<3∑u′∈S2dudu′dS+∑u∈T∑u′∈Sdu′<32dudTdu′+∑u∈Tdu≥3∑u′∈Sdu′≥3dudTdu′dS≤2(dT+dS)∑u∈Tdu∑u′∈Sdu′+dTdS∑u∈Tdu≥3du∑u′∈Sdu′≥3du′=Lem.3O((dT+dS)nTnS+dTdSLTLS).

Thus, the overall *MCM* computation time due to term **III** in Equation 7 is *O*((*d*_*T*_ + *d*_*S*_)*n*_*T*_*n*_*S*_ + *d*_*T*_*d*_*S*_*L*_*T*_*L*_*S*_ + min(*d*_*T*_,*d*_*S*_)^**3**^*L*_*T*_*L*_*S*_).

Equation 5 is computed once for each *v* ∈ *T* and *v*′ ∈ *S* in line 3, where the corresponding matching instance’s group sizes are *d*_*v*_ and$d_{v\prime }$. Similarly as above, it can be shown that summing the total number of operations in the implied *MCM* computations yields

$$\;\underset{u\in T}{\sum }\underset{u^{\prime }\in S}{\sum }\left(\text{min}{\left(d_{u},d_{u^{\prime }}\right)}^{3}+d_{u}d_{u^{\prime }}\right)\overset{\text{Lem.3}}{=}O(n_{T}n_{S}+\text{min}(d_{T},d_{S})L_{T}L_{S}).$$

Therefore, the overall running time of Algorithm 1 is dictated by the the bottleneck expression *O*((*d*_*T*_ + *d*_*S*_)*n*_*T*_*n*_*S*_ + *d*_*T*_*d*_*S*_*L*_*T*_*L*_*S*_ + min(*d*_*T*_,*d*_*S*_)^3^*L*_*T*_*L*_*S*_).

### Improving the time complexity

The time analysis in the previous section shows that all operations in Algorithm 1, besides *MCM* computations due to term **III** of Equation 7, are conducted in *O*(*n*_*T*_*n*_*S*_ + min(*d*_*T*_,*d*_*S*_)*L*_*T*_*L*_*S*_) time. In this section, we show how to improve the time complexity of Algorithm 1, by incorporating *cavity matching* subroutines to speed up the *MCM* computations due to term **III** of Equation 7.

**Figure 7:**
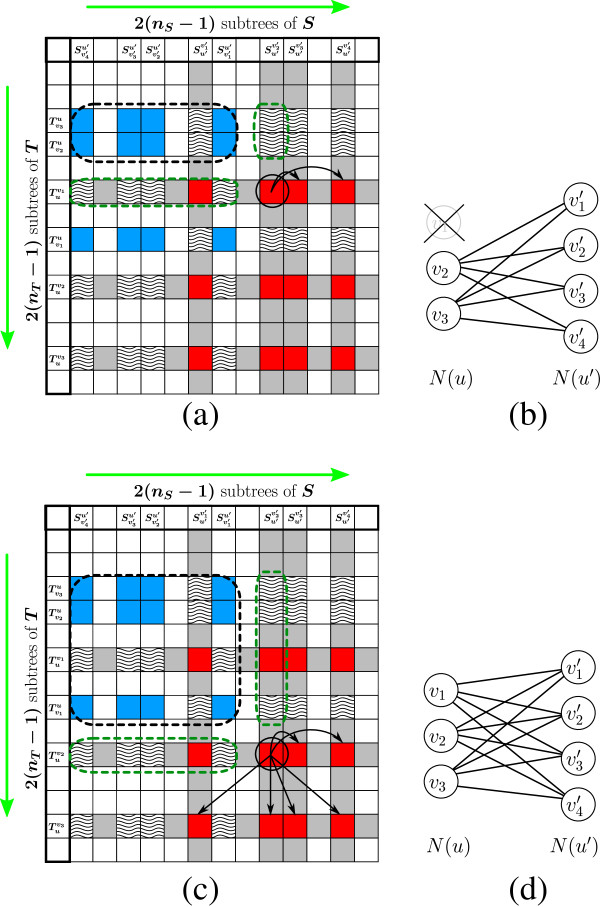
**The incorporation of cavity matching subroutines in the DP algorithm.** (**a**) Upon reaching the entry corresponding to$T_{u}^{v_{1}}$ and$S_{u^{\prime }}^{v_{2}^{\prime }}$, all values of the form${\text{Rooted-HSA}}^{-r}(T_{v_{j}}^{u},S_{v_{j^{\prime }}^{\prime }}^{u^{\prime }})$ for *v*_*j*_ ∈ *N*(*u*) ∖ {*v*_1_} and$v_{j^{\prime }}^{\prime }\in N(u^{\prime })$ are computed (see (**b**)). This allows to compute all values$\text{MCM}(N(u)\setminus \{v_{1}\},N(u^{\prime })\setminus \{v_{j}^{\prime }\},w_{u,u^{\prime }}^{v_{1},v_{j}^{\prime }})$, by solving the *All*-*Cavity*-*MCM* problem over the instance$\left(N(u)\setminus \{v_{1}\},N(u^{\prime }),w_{u,u^{\prime }}^{v_{1}}\right)$, and thus computing term **III** with respect to all remaining entries in the first row of$H_{u,u^{\prime }}$. (**c**) Upon reaching the entry corresponding to$T_{u}^{v_{2}}$ and$S_{u^{\prime }}^{v_{2}^{\prime }}$, all values of the form${\text{Rooted-HSA}}^{-r}(T_{v_{j}}^{u},S_{v_{j^{\prime }}^{\prime }}^{u^{\prime }})$ for *v*_*j*_ ∈ *N*(*u*) and$v_{j^{\prime }}^{\prime }\in N(u^{\prime })$ are computed (see (**d**)). This allows to compute all values$\text{MCM}(N(u)\setminus \{v_{j}\},N(u^{\prime })\setminus \{v_{j^{\prime }}^{\prime }\},w_{u,u^{\prime }}^{v_{j},v_{j^{\prime }}^{\prime }})$, by solving the *All*-*Pairs*-*Cavity*-*MCM* problem over the instance$\left(N(u),N(u^{\prime }),w_{u,u^{\prime }}\right)$, and thus computing term **III** with respect to all remaining entries in$H_{u,u^{\prime }}$.

Let *u* ∈ *T*, *u*′ ∈ *S*, and consider the computation of term **III** of Equation 7 for instances in the first row in$H_{u,u^{\prime }}$. Note that for the entries in this row, the first group in the bipartite matching instance is fixed and equals to *N*(*u*) ∖ {*v*_**1**_}, whereas for each column *j*, the second group in the matching instance is$N(u^{\prime })\setminus \{v_{j}^{\prime }\}$. The first entry in this row is computed by solving the *MCM* problem directly for the matching instance$\left(N(u)\setminus \{v_{1}\},N(u^{\prime })\setminus \{v_{1}^{\prime }\},w_{u,u^{\prime }}^{v_{1},v_{1}^{\prime }}\right)$ (Figures 6a,6b). Based on Observation 2, upon reaching the second entry in this row, all solutions${\text{Rooted-HSA}}^{-r}\left(T_{v_{i}}^{u},S_{v_{j}^{\prime }}^{u^{\prime }}\right)$ for *i* > 1 and *j* ≥ 1 are computed and stored in *H*. Therefore, the *All*-*Cavity*-*MCM* problem can be solved for the matching instance$\left(N(u)\setminus \{v_{1}\},N(u^{\prime }),w_{u,u^{\prime }}^{v_{1}}\right)$, where$w_{u,u^{\prime }}^{v_{1}}$ is defined similarly as$w_{u,u\prime }$ with respect to the sets *N*(*u*)∖{*v*_**1**_} and *N*(*u*′). This allows to compute term **III** for each one of the remaining entries in this row of$H_{u,u^{\prime }}$ in *O*(1) time (Figures 7a,7b).

The first entry of the second row in$H_{u,u^{\prime }}$ is again computed directly by solving the *MCM* problem for the matching instance$\left(N(u)\setminus \{v_{2}\},N(u^{\prime })\setminus \{v_{1}^{\prime }\},w_{u,u^{\prime }}^{v_{2},v_{1}^{\prime }}\right)$. Upon reaching the second entry of the second row of$H_{u,u^{\prime }}$, Observation 2 implies that all solutions${\text{Rooted-HSA}}^{-r}\left(T_{v_{i}}^{u},S_{v_{j}^{\prime }}^{u^{\prime }}\right)$ for *i*,*j* ≥ 1 are already computed and stored in *H*. Therefore, the *All*-*Pairs*-*Cavity*-*MCM* problem can be solved for the matching instance$\left(N(u),N(u^{\prime }),w_{u,u^{\prime }}\right)$, allowing to compute term **III** for each one of the remaining entries in$H_{u,u^{\prime }}$ in *O*(1) time (Figures 7c,7d). Thus, computing term **III** for all entries in$H_{u,u^{\prime }}$ is done by solving the *MCM* problem twice, solving the *All*-*Cavity*-*MCM* problem once, and solving the *All*-*Pairs*-*Cavity*-*MCM* problem once, where the sizes of the two groups in the matching instances for these problems are at most *d*_*u*_ and$d_{u^{\prime }}$. Based on Theorem 1, this whole *MCM* computation for sub- matrix$H_{u,u^{\prime }}$ takes$O(\text{min}{\left(d_{u},d_{u^{\prime }}\right)}^{3}+d_{u}d_{u^{\prime }})$ time. Recall that matrix *H* is decomposed into matrices$H_{u,u^{\prime }}$ for all pairs *u* ∈ *T*, *u*′ ∈ *S*, and so the total computation time of term **III** in Equation 7 throughout the entire run of the algorithm is

$$\underset{u\in T}{\sum }\underset{u^{\prime }\in S}{\sum }\left(\text{min}{\left(d_{u},d_{u^{\prime }}\right)}^{3}+d_{u}d_{u^{\prime }}\right)\overset{\text{Lem.3}}{=}O(n_{T}n_{S}+\text{min}(d_{T},d_{S})L_{T}L_{S}),$$

matching the running time of all other computations, and we get the following theorem:

#### 

**Theorem 2.** Algorithm 1 can be implemented with an *O*(*n*_*T*_*n*_*S*_ + min(*d*_*T*_,*d*_*S*_)*L*_*T*_*L*_*S*_) = *O*(min(*n*_*T*_,*n*_*S*_) *n*_*T*_*n*_*S*_) time complexity.

We would like to emphasize that replacing *n*_*T*_ and *n*_*S*_ by *L*_*T*_ and *L*_*S*_ or *d*_*T*_ and *d*_*S*_ in the time complexity term of Theorem 2 is due to a refined analysis rather than some algorithmic improvement, and such an analysis can also be applied to refine the time complexities of some of the previous algorithms. While in many tree comparison applications typical input trees *T* are characterized by having the number of leaves at the same order of magnitude as the number of nodes (i.e. *L*_*T*_ **=** *Θ* (*n*_*T*_)), and sometimes it is true that *d*_*T*_ **=***Θ* (*n*_*T*_) (e.g. in star-like trees), there are cases where *L*_*T*_,*d*_*T*_ ≪ *n*_*T*_. Specifically, it can be asserted that removing (by node smoothing) or adding (by subdividing edges) degree-2 nodes to a tree do not change its maximum degree nor the number of its leaves, and thus trees with a high number of degree-2 nodes have a low maximum degree and a small number of leaves with respect to the total number of nodes. As can be shown in our examples (see Section ‘RNA tree representation’), typical RNA trees in our application do have a relatively high number of degree-2 nodes, and thus gain from the fact that the cubic term in the time complexity of the algorithm depends on the maximum node degrees and the number of leaves, rather than the number of nodes in the input trees.

## Algorithms for bipartite matching problems

In this section we show efficient algorithms for the *MCM*, *All-Cavity-MCM*, and *All-Pairs-Cavity-MCM* problems defined in Section ‘Min-Cost bipartite matching’. These algorithms are based on a reduction to the *Min-Cost Max-Flow* problem. Since the *Min-Cost Max-Flow* problem is well known to computer scientists, we only provide a brief discussion of essential properties required for describing our algorithms, while omitting some of the details. For a definition of the *Min-Cost Max-Flow* problem, related theorems, and a thorough discussion of its properties, please refer to other works, e.g. [30,39,40].

Throughout this section, let (*X*,*Y*,*w*) be a matching instance. Assume w.l.o.g. that |*X*| ≤ |*Y*|, and denote |*X*| = *n*,|*Y*| = *m*. When the context is clear, instead of writing a “matching *M* for (*X*,*Y*,*w*)”, we simply write a “matching *M*”, and similarly when writing “*M* is an optimal matching” we mean that *M* is optimal with respect to (*X*,*Y*,*w*).

### **Efficient algorithm for *MCM***

Next, we refine the reduction of *MCM* to *Min-Cost Max-Flow* given at [27], in order to to improve the running time of the algorithm. Our modification reduces the *O*(*nm*^**2**^ **+** *m*^**2**^ log*m*) running time of the algorithm of [27] to *O*(*n*^3^ + *nm*), using a variant of the approach of [29].

#### ***Reducing MCM to Min-Cost Max-Flow***

For *x* ∈ *X* and *y* ∈ *Y*, define the *effective matching costw*_*e*_(*x*,*y*) of the pair (*x*,*y*) to be *w*_*e*_(*x*,*y*) = *w*(*x*,*y*) - *w*(*x*) - *w*(*y*). The effective matching cost *w*_*e*_(*x*,*y*) is the cost change due to the addition of the pair (*x*,*y*) into a matching in which both *x* and *y* are unmatched.

For each *x* ∈ *X*, define a subset *Y*_*x*_ ⊆ *Y* of *n* smallest cost matches for *x* in *Y*, with respect to the effective matching costs. Define$Y_{X}=\underset{x\in X}{⋃}Y_{x}$.

##### 

**Lemma 4.** There exists an optimal matching *M*^∗^ such that *M*^∗^ ⊆ *X* × *Y*_*X*_.

##### 

*Proof.* Let *M*^∗^ be an optimal matching. Since each element in *X* participates in at most one pair in *M*^∗^, *M*^∗^ contains at most *n* pairs, and so there are at most *n* distinct elements *y* ∈ *Y* such that *y* ∈ *M*. If *M*^∗^ contains a pair (*x*,*y*) such that *y* ∉ *Y*_*X*_, then *y* ∉ *Y*_*X*_, and in particular there must be some *y*′ ∈ *Y*_*x*_ such that *y*′ ∉ *M* (since |*Y*_*x*_| = *n*). By definition of *Y*_*x*_, *w*_*e*_(*x*,*y*′) ≤ *w*_*e*_(*x*,*y*), and the matching *M*′∗ obtained by removing the pair (*x*,*y*) from *M*^∗^ and adding the pair (*x*,*y*′) satisfies *w*(*M*′∗) = *w*(*M*^∗^) - *w*_*e*_(*x*,*y*) + *w*_*e*_(*x*,*y*′) ≤ *w*(*M*^∗^). Since *M*^∗^ is optimal, *M*′∗ is also optimal. It is possible to continue and apply such modifications until getting an optimal matching *M*′′^∗^ which contains only pairs (*x*,*y*) such that *y* ∈ *Y*_*X*_, as required. □

**Figure 8:**
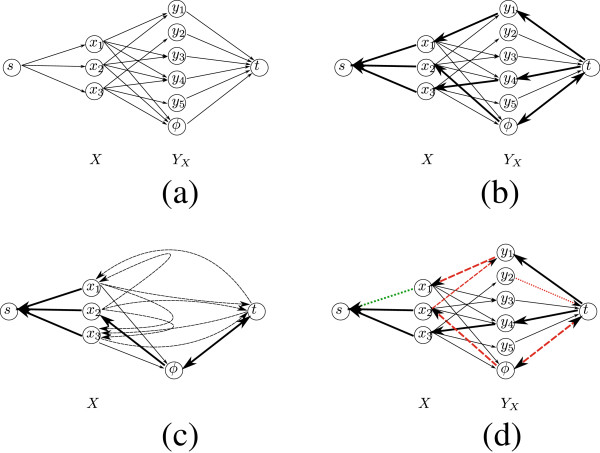
**The reduction from*MCM*to*Min-Cost Max-Flow*.** (a) The graph *G* constructed in the case where |*X*|=3 and |*Y*_*X*_| = 5. All edge capacities are 1, except for the edge (*ϕ*,*t*) whose capacity is *c*(*ϕ*,*t*) = |*X*| = 3. Edges of the form (*x*_*i*_,*y*_*j*_) have the costs *w*_*e*_(*x*_*i*_,*y*_*j*_) = *w*(*x*_*i*_,*y*_*j*_) - *w*(*x*_*i*_) - *w*(*y*_*j*_), and all other edges have zero costs. (b) The residual graph *G*^*f*^ after finding a minimum cost maximum flow *f* in the network. Edges over which there is a flow (depicted as thickened edges) reverse their direction (the edge (*ϕ*,*t*) is not saturated, and therefore both (*ϕ*,*t*) and (*t*,*ϕ*) belong to the residual graph). In this example, the flow implies the minimum cost matching *M*_*f*_ = {(*x*_**1**_,*y*_**1**_),(*x*_**3**_,*y*_**4**_)}, where the elements *x*_**2**_,*y*_**2**_,*y*_**3**_, and *y*_**5**_ are unmatched. (**c**) The graph${Ĝ}^{f}$ obtained from *G*^*f*^. All nodes in the set *Y*_*x*_ are removed, and length-2 paths of the form *u* → *y* → *v* are replaced with direct edges (*u*,*v*) (these are edges in the set${Ê}_{2}^{f}$, depicted with dashed lines in the figure). The cost of an edge (*u*,*v*) is the minimum among the costs of the corresponding length-2 paths from *u* to *v* through a node in *Y*_*x*_. Finding minimum cost paths from *s* to all nodes in${Ĝ}^{f}$ can be done in *O*(*n*^**2**^) time, where in additional *O*(*n*^**2**^) operations it is possible to obtain minimum cost paths from *s* to all nodes in *G*^*f*^. (**d**) A minimum cost path from *y*_**2**_ to *x*_**1**_ in *G*^*f*^. The path, depicted in dashed and dotted red, is *P* = *y*_**2**_ → *t* → *ϕ* → *x*_2_ → *y*_1_ → *x*_1_. The corresponding return path *P*^′^** = ***t* → *ϕ* → *x*_**2**_** → ***y*_**1**_** → ***x*_**1**_** → ***s* from *t* to *s* is obtained by removing from *P* the first edge *y*_**2**_** → ***t*v(in dotted red), and adding at the end the edge *x*_**1**_** → ***s* (in dotted green). The flow *f*^′^vobtained by returning one flow unit from *t* to *s*  along *P*^′^ is an optimal flow in the network$N_{x_{1},y_{2}}$, corresponding to the sub-instance (*X* ∖ {*x*_**1**_},*Y* ∖ {*y*_**2**_},*w*) (see proof of Lemma 10). The corresponding matching for this flow is$M_{f^{\prime }}=\{(x_{2},y_{1}),(x_{3},y_{4})\}$, which is an optimal matching for (*X* ∖ {*x*_**1**_},*Y* ∖ {*y*_**2**_},*w*).

Next, we describe how to reduce *MCM* into the Min-Cost Max-Flow problem. The reduction builds the cost flow network *N* = (*G*,*s*,*t*,*c*), where *G* is the network’s weighted graph, *s* and *t* are the source and sink nodes respectively, and *c* is the edge capacity function. The graph *G* = (*V*,*E*) is defined as follows (Figure 8a): 

• *V* = *X* ∪ *Y*_*X*_∪{*s*,*t*,*ϕ*}, where *s*, *t*, and *ϕ* are unique nodes different from all nodes in *X* and *Y*_*x*_. Note that we use the same notations for elements in *X* and *Y*_*x*_ and their corresponding nodes in *V*, where ambiguity can be resolved by the context. By definition, |*Y*_*X*_| ≤ *n*^2^, and so |*V*| = *O*(*n*^2^).

• *E* = *E*_**1**_ **∪** *E*_**2**_, where *E*_**1**_ **=** {(*x*,*y*):*x* ∈ *X*,*y* ∈ *Y*_*x*_}, and *E*_**2**_ **=** {(*s*,*x*):*x* ∈ *X*} ∪ {(*y*,*t*):*y* ∈ *Y*_*X*_} ∪ {(*x*,*ϕ*):*x* ∈ *X*} ∪ {(*ϕ*,*t*)}. The cost *cost*(*x*,*y*) of every edge (*x*,*y*) ∈ *E*_**1**_ is the corresponding effective matching cost *w*_*e*_(*x*,*y*), and the cost *cost*(*u*,*v*) of each edge (*u*,*v*) ∈ *E*_**2**_ is zero. Note that |*E*| = *O*(*n*^**2**^.

The capacity function *c* assigns unit capacities to all edges in *E*, with the exception that *c*(*ϕ*,*t*) = *n*.

For a maximum flow *f* in *N*, define the set of pairs *M*_*f*_ **=** {(*x*,*y*) : *x* ∈ *X*,*y* ∈ *Y*_*X*_,*f*(*x*,*y*) = 1}.From flow conservation constraints, it is simple to assert that every *z* ∈ *X* ∪ *Y* participates in at most one pair in *M*_*f*_, and thus *M*_*f*_ is a valid matching, and in addition, *M*_*f*_ **⊆** *X* × *Y*_*X*_. For a matching *M* for (*X*,*Y*,*w*) such that *M* ⊆ *X* × *Y*_*X*_, define the maximum flow *f*_*M*_ in *N* as the flow which is obtained by transmitting one flow unit on every path of the form *s* → *x* → *y* → *t* for all (*x*,*y*) ∈ *M*, and one flow unit on every path of the form *s* → *x* → *ϕ* → *t* for all *x* ∈ *X* such that *x* ∉ *M*. It is simple to observe that *f*_*M*_ is a valid flow in *N* (satisfying the capacity and flow conservation constraints), where its maximality is asserted from the fact that it saturates the cut ({*s*},*V* ∖ {*s*}). Note that for every matching *M* ⊆ *X* × *Y*_*X*_,$M_{f_{M}}=M$, and for every maximum flow *f* in *N*,$f_{M_{f}}=f$.

Denote by *cost*(*f*) the cost of a flow *f* in *N*, and by *w*_*X*,*Y*_ the summation$w_{X,Y}=\underset{z\in X\cup Y}{\sum }w(z)$.

Lemma 5 shows the relation between a cost of a matching *M* ⊆ *X* × *Y*_*X*_ and its corresponding flow *f*_*M*_.

##### 

**Lemma 5.** For every matching *M* ⊆ *X* × *Y*_*X*_, *w*(*M*) = *cost*(*f*_*M*_) + *w*_*X*,*Y*_.

##### 

*Proof.* Note that only edges in *E*_**1**_ in *N* are assigned nonzero costs, and therefore the cost of *f*_*M*_ is given by$\text{cost}(f_{M})=\underset{(x,y)\in E_{1}}{\sum }f_{M}(x,y)\cdot \text{cost}(x,y)$. In addition, note that for every (*x*,*y*) ∈ *E*_**1**_, *f*_*M*_(*x*,*y*) = 1 if (*x*,*y*) ∈ *M*, and otherwise *f*_*M*_(*x*,*y*) = 0.

Hence,$\text{cost}(f_{M})\;=\;\underset{(x,y)\in M}{\sum }\text{cost}(x,y)=\underset{(x,y)\in M}{\sum }w_{e}(x,y)$. Now,

w(M)=∑(x,y)∈Mw(x,y)+∑z∈X∪Yz∉Mw(z)=∑(x,y)∈Mwe(x,y)+w(x)+w(y)+∑z∈X∪Yz∉Mw(z)=∑(x,y)∈Mwe(x,y)+∑z∈X∪Yz∈Mw(z)+∑z∈X∪Yz∉Mw(z)=cost(fM)+wX,Y.

□

Call a minimum-cost maximum-flow in *N* an *optimal* flow with respect to *N*. Proposition 1 concludes the relation between optimal flows in *N* and optimal matchings of (*X*,*Y*,*w*).

##### 

**Proposition 1.** Let *f*^**∗**^ an optimal flow in *N*. Then,$\text{MCM}(X,Y,w)=w(M_{f^{*}})=\text{cost}(f^{*})+w_{X,Y}$.

##### 

*Proof.* For the matching$M_{f^{*}}$, we have that *MCM*$(X,Y,w)\leq w(M_{f^{*}})\overset{\text{Lem.5}}{=}\text{cost}(f_{M_{f^{*}}})+w_{X,Y}=\text{cost}(f^{*})+w_{X,Y}$. On the other hand, from Lemma 4 there is an optimal matching *M*^**∗**^ such that *M*^**∗**^ **⊆** *X* × *Y*_*X*_, and$\text{MCM}(X,Y,w)=w(M^{*})\overset{\text{Lem.5}}{=}\text{cost}(f_{M^{*}})+w_{X,Y}\geq \text{cost}(f^{*})+w_{X,Y}$, proving the proposition. □

#### ***Efficient computation and time complexity***

For an efficient computation of an optimal flow in *N*, we first describe a modification that can be applied to residual graphs and allows a computational speed up.

Let *f* be a flow in *N*, and *y* ∈ *Y*_*X*_. Due to the flow conservation constraints, either *f* does not transmit any flow unit through *y*, or *f* transmits one flow unit that enters *y* over some ingoing edge (*x*,*y*) (where *y* ∈ *Y*_*X*_), and leaves *y* by the unique outgoing edge (*y*,*t*) in *E*.In both cases, and since all edges adjacent to *y* have a unit capacity, the residual graph *G*^*f*^ **=** (*V*,*E*^*f*^) contains a single outgoing edge from *y*: the original edge (*y*,*t*) in the former case, or the residual edge (*y*,*x*) in the latter case (where the edge (*y*,*t*) is reversed in *G*^*f*^ to become an ingoing edge (*t*,*y*)). Denote by *v*_*y*_ the unique node in *X* ∪ {*t*} into which there is an outgoing edge from *y* in *E*^*f*^. In addition, observe that the number of ingoing edges into *y* in *G*^*f*^ equals to the number of ingoing edges into *y* in *G*, implying the following observation:

##### Observation 3

$\underset{y\in Y_{X}}{\sum }\;\;\left|\left\{(u,y)\in E^{f}\right\}\right|\;=\;\;\underset{y\in Y_{X}}{\sum }\left|\left\{(u,y)\in E\right\}\right|\;=\;\left|E_{1}\right|\;=\;n^{2}.$

Let${Ĝ}^{f}=(\hat{V},{Ê}^{f})$ be the graph defined by 

• $\hat{V}=X\cup \{s,t,\phi \}$,

• ${Ê}^{f}={Ê}_{1}^{f}\cup {Ê}_{2}^{f}$, where 

${Ê}_{1}^{f}=E^{f}\setminus \left\{(u,v)\in E^{f}:u\in Y_{X}\text{or}\;v\in Y_{X}\right\}$, and

${Ê}_{2}^{f}=\left\{(u,v):\exists y\in Y_{X}\text{s.t.}(u,y)\in E^{f}\text{and}\;v=v_{y}\right\}$.

Observe that${Ê}_{1}^{f}$ is included in *E*^*f*^. Denote by$\text{cos}t_{{Ê}^{f}}$ the cost function over edges in${Ê}^{f}$. For$(u,v)\in {Ê}_{1}^{f}$ define$\text{cos}t_{{Ê}^{f}}(u,v)=\text{cost}(u,v),$ and for$(u,v)\in {Ê}_{2}^{f}$ define$\text{cos}t_{{Ê}^{f}}(u,v)=\underset{(}{\text{min}}\text{cost}(u,y)+\text{cost}(y,v)).$*y* ∈ *Y*_*X*_ : (*u*,*y*) ∈ *E*^*f*^ and *v* = *v*_*y*_.

For nodes *u*,*v* ∈ *V*, denote by$d_{u,v}^{f}$ the minimum cost of a path from *u* to *v* in *G*^*f*^, and for$u,v\in \hat{V}$, denote by${\hat{d}}_{u,v}^{f}$ the minimum cost of a path from *u* to *v* in${Ĝ}^{f}$ (if there is no path between *u* and *v* in one of these graphs, define the corresponding minimum path cost to be *∞*).

##### 

**Lemma 6.** For every$u,v\in \hat{V}$,$d_{u,v}^{f}={\hat{d}}_{u,v}^{f}$.

##### 

*Proof.* Let$u,v\in \hat{V}$, and let *P* be a minimum cost path in *G*^*f*^ from *u* to *v*. If *P* traverses a node *y*′ ∈ *Y*_*x*_, this traversal is of the form *u*^′^ → *y*^′^ → *v*^′^ for some$u^{\prime },v^{\prime }\in \hat{V}$ such that (*u*^′^,*y*^′^**) ∈ ***E*^*f*^ and$v^{\prime }=v_{y^{\prime }}$ (note that there are no edges between two nodes in *Y*_*x*_). By construction, the edge (*u*^′^,*v*^′^) is in${Ê}_{2}^{f}$, where its cost satisfies$\text{cos}t_{{Ê}^{f}}(u^{\prime },v^{\prime })=\underset{(}{\text{min}}\text{cost}(u^{\prime },y)+\text{cost}(y,v^{\prime }))\leq \text{cost}(u^{\prime },y^{\prime })+\text{cost}(y^{\prime },v^{\prime })$.*y* ∈ *Y*_*X*_ : (*u*^′^,*y*) ∈ *E*^*f*^ and *v*^′^ = *v*_*y*_.

Thus, each such sub-path *u*′→*y*′→*v*′ in *P* can be replaced by an edge$(u^{\prime },v^{\prime })\in {Ê}_{2}^{f}$, where the cost of the replacing edge is at most the cost of the sub-path. This yields a corresponding path$\hat{P}$ from *u* to *v* in${Ĝ}^{f}$, which has the same or lower cost than *P*. In particular,$d_{u,v}^{f}\geq {\hat{d}}_{u,v}^{f}$.

On the other hand, let$\hat{P}$ be a minimum cost path in${Ĝ}^{f}$ from *u* to *v*. Similarly as above, every edge$(u^{\prime },v^{\prime })\in {Ê}_{2}^{f}$ in $\hat{P}$ can be replaced by a path *u*′ → *y* → *v*′ in *E*^*f*^ such that $\text{cost}(u^{\prime },y)+\text{cost}(y,v^{\prime })=\text{cos}t_{{Ê}^{f}}(u^{\prime },v^{\prime })$, yielding a path *P* from *u* to *v* in *G*^*f*^ of the same cost as $\hat{P}$. Hence $d_{u,v}^{f}\leq {\hat{d}}_{u,v}^{f}$, and so we get that if there is a path from *u* to *v* in one of the graphs,$d_{u,v}^{f}={\hat{d}}_{u,v}^{f}$. In the case where there are no paths from *u* to *v* in both graphs,$d_{u,v}^{f}={\hat{d}}_{u,v}^{f}=\infty$. □

##### 

**Lemma 7.** For every *y* ∈ *Y*_*X*_ and every$v\in \hat{V}$,$d_{y,v}^{f}=\text{cost}(y,v_{y})+{\hat{d}}_{v_{y},v}^{f}$, and$d_{v,y}^{f}=\underset{(u,y)\in E^{f}}{\text{min}}({\hat{d}}_{v,u}^{f}+\text{cost}(u,y))$.

##### 

*Proof.* Let *y* ∈ *Y*_*X*_ and$v\in \hat{V}$. A minimum cost path *P* from *y* to *v* in *G*^*f*^ (if there is such a path) must start with the only outgoing edge (*y*,*v*_*y*_) from *y*, and from the optimality of *P* the remainder of *P* is a minimum cost path from *v*_*y*_ into *v* in *G*^*f*^. Thus, the cost of *P* is $d_{y,v}^{f}=\text{cost}(y,v_{y})+d_{v_{y},v}^{f}\overset{\text{Lem.6}}{=}\text{cost}(y,v_{y})+{\hat{d}}_{v_{y},v}^{f}$. If there is no path from *y* to *v* in *G*^*f*^, then in particular there is no path from *v*_*y*_ to *v* in *G*^*f*^, and$d_{y,v}^{f}=\infty =d_{v_{y},v}^{f}\overset{\text{Lem.6}}{=}{\hat{d}}_{v_{y},v}^{f}=\text{cost}(y,v_{y})+{\hat{d}}_{v_{y},v}^{f}$.

To show the second equality in the lemma, observe similarly that for a minimum cost path *P* from *v* to *y* in *G*^*f*^ and the last edge (*u*′,*y*) in *P*, the cost of *P* is$d_{v,y}^{f}=d_{v,u^{\prime }}^{f}+\text{cost}(u^{\prime },y)\overset{\text{Lem.6}}{=}{\hat{d}}_{v,u^{\prime }}^{f}+\text{cost}(u^{\prime },y)\geq \underset{(u,y)\in E^{f}}{\text{min}}({\hat{d}}_{v,u}^{f}+\text{cost}(u,y))$. Since for every *u* such that (*u*,*y*) ∈ *E*^*f*^ there is a path from *v* to *y* of cost$d_{v,u}^{f}+\text{cost}(u,y)$ (the path concatenating the edge (*u*,*y*) at the end of a minimum cost path from *v* to *u* in *G*^*f*^), it follows that$d_{v,y}^{f}\;\leq \;\underset{\;(u,y)\in E^{f}}{\text{min}}\;(\;d_{v,u}^{f}\;+\;\text{cost}(\;u,\;y\;)\;)\;\overset{\text{Lem.6}}{=}\;\underset{(u,y)\in E^{f}}{\text{min}}\;({\hat{d}}_{v,u}^{f}\;+\;\text{cost}(\;u,\;y\;)\;)$, and we get that$d_{v,y}^{f}=\underset{(u,y)\in E^{f}}{\text{min}}({\hat{d}}_{v,u}^{f}+\text{cost}(u,y))$. The case where there is no path from *v* to *y* in *G*^*f*^ is resolved similarly as above. □

Using Lemmas 6 and 7, we turn to analyze the time complexity for the reduction we described for solving *MCM*(*X*,*Y*,*w*).

The computation of each subset *Y*_*x*_ can be done in *O*(*m*) time, using the algorithm of [41], and so *Y*_*x*_ can be computed in *O*(*nm*) time. The network *N* contains *O*(*n*^2^) nodes and edges, and thus can be constructed in *O*(*n*^2^) time.

An optimal flow in *N* can be found with the algorithm of Edmonds and Karp [30]. Essentially, this is an iterative algorithm that maintains a valid flow *f* in *N*. Starting with a zero-flow, at each iteration the algorithm increases the flow by adding the bottleneck capacity flow along a minimum cost path from *s* to *t* in the residual graph *G*^*f*^. In our specific reduction, all edges leaving *s* have a unit capacity, and thus each augmentation path increases the size of the flow by one unit. As the size of the maximum flow in *N* is *n* (asserted by the minimum cut ({*s*},*V* ∖ {*s*}) of capacity *n* in *N*), the algorithm performs *n* iterations.

The time required for each iteration is dictated by the time required for computing minimum cost paths from *s* into every node in the residual graph *G*^*f*^. Such paths can be computed efficiently for weighted graphs with nonnegative edge costs using Dijkstra’s algorithm [42]. In order to render all edge costs in the residual graphs to nonnegative, the algorithm of [30] applies a *node labeling* function. Such a function assigns to each node *v* ∈ *V* a label *l*(*v*), and shortest paths in the residual network are computed with respect to the modified edge costs *cost*′(*u*,*v*) = *cost*(*u*,*v*) + *l*(*u*) - *l*(*v*). It is known that a path from *u* to *v* in *G*^*f*^ is of minimum cost with respect to the original cost function if and only if it is of minimum cost with respect to the modified cost function, and that setting$l(v)=d_{s,v}^{f}$ for every *v* ∈ *V*, where *f* is the flow at the beginning of the *i*-th iteration, guarantees nonnegative modified edge costs in the residual graph at the beginning of the (*i*+1)-th iteration (after *f* was augmented and the residual graph was modified, see [30]). Thus, given that minimum cost paths are computed from *s* to all nodes in the residual graph, label maintenance at each iteration is done in linear time with respect to the number of nodes in the network.

In order to compute minimum cost paths from *s* to all nodes in *G*^*f*^, we first construct the corresponding graph${Ĝ}^{f}$ as described above (under the assumption that edge costs are rendered to be nonnegative). It is simple to observe that this construction can be implemented in *O*(*n*^2^) time. By construction,$\left|\hat{V}|=\left|X\cup \{s,t,\phi \}\right|=O(n\right)$. Using Dijkstra’s algorithm [42],${\hat{d}}_{s,v}^{f}$ can be computed for all$v\in \hat{V}$ in$O\left(|\hat{V}{|}^{2}\right)=O(n^{2})$ time. Note that we are referring to the original Dijkstra algorithm (published in 1959) rather than to its commonly used improvement due to Fredman and Tarjan [31]. While the latter improvement reduces the running time of the algorithm for non-dense graphs, it involves the usage of a relatively sophisticated data structure (Fibonacci heap). In our case, the simple implementation described in [42] does not exceed the *O*(*n*^**2**^) running time required for the construction of${Ĝ}^{f}$, without the usage of any kind of priority queue or other complex data structures.

Given the values${\hat{d}}_{s,v}^{f}$ for all$v\in \hat{V}$, values$d_{s,v}^{f}$ can be computed for all *v* ∈ *V* by applying Lemmas 6 and 7. The time required for computing distances$d_{s,v}^{f}$ for all$v\in \hat{V}=V\setminus Y_{X}$ due to Lemma 6 is$O\left(\left|\hat{V}\right|\right)=O(n)$, and the time required for computing distances$d_{s,y}^{f}$ for all *y* ∈ *Y*_*X*_ due to Lemma 7 is$\underset{y\in Y_{X}}{\sum }\left|\left\{(u,y)\in E^{f}\right\}\right|\overset{\text{Obs.3}}{=}O(n^{2})$. Therefore, each iteration is conducted in *O*(*n*^2^) time, and the total running time of all *n* iterations of the algorithm is *O*(*n*^3^).

A special attention is required though for the initialization of the algorithm of [30]. There, it was assumed that all edges in the input network *N* are of nonnegative costs, while our described reduction from *MCM* does not sustain this property. Nevertheless, this may be overcome by setting the initial labels of all nodes *v* ∈ *V* to the corresponding minimum path costs *d*_*s*,*v*_ in the graph *G* of *N*. Since *G* is acyclic, these initial costs can be computed in *O*(|*V*| + |*E*|) = *O*(*n*^2^) time using a simple topological traversal (see e.g., [43]).

In all, we get that the total running time required for constructing a flow network *N* corresponding to the matching instance (*X*,*Y*,*w*) and finding an optimal flow in *N* is *O*(*n*^3^ + *nm*). Computing *w*_*X*,*Y*_ can be done in *O*(*n* + *m*) time, and applying Proposition 1, *MCM*(*X*,*Y*,*w*) can be computed in *O*(*n*^3^ + *nm*) time, proving the statement in Theorem 1 regarding *MCM*.

#### ***Additional practical improvements using sparsification***

It is possible to further improve the running time of the reduction in practice, by reducing the number of edges in the network, and computing *Min-Cost Flow* instead of Min-Cost Max-Flow.

We may assume without lost of generality that an optimal matching *M*^∗^between *X* and *Y* contains no pairs (*x*,*y*) such that *w*(*x*,*y*) - *w*(*x*) - *w*(*y*) ≥ 0 (since removing such an edge from the matching can only decrease its cost). Consequentially, there is an optimal flow in *N* that does not transmit flow through edges (*x*,*y*) for which *w*′(*x*,*y*) = *w*(*x*,*y*) - *w*(*x*) - *w*(*y*) ≥ 0. Therefore, the cost of an optimal flow in the network *N*′, obtained by removing such edges from *N*, equals to the cost of an optimal flow in *N*. Note that in the case of RNA tree alignment, removed edges correspond to pairs of subtrees which are sufficiently dissimilar so that their pruning cost is lower than their alignment cost. For a reasonable cost function, it is expected that many of the subtree-pairs of the input would sustain this condition (even in the extreme case of aligning a tree to itself), as they represent different parts of complex molecules.

The second improvement exploits a property of the Min-Cost Max-Flow algorithm of [30], for which it was shown that the series of computed augmentation paths are of non-decreasing costs (Theorem 5 in [30]). Note that as long as there is an augmentation path in the network, there is such a path with cost zero (since there is some node *x* for which (*s*,*x*) is not saturated, and thus the path *s* → *x* → *ϕ* →*t* is an augmentation path of cost zero). Therefore, once the minimum cost of an augmentation path is zero, all consecutive augmentation paths would have a zero cost, and increasing the flow along these paths will not change the overall cost of the flow. It is thus possible to stop the flow algorithm upon the first iteration at which the minimum augmentation path cost is zero, where the flow at this stage is a minimum-cost flow which is not necessarily of maximum size (while it has the same cost as a min-cost max-flow). It is possible to show that increasing the flow over a negative cost augmentation path necessarily increases the size of the corresponding matching (otherwise it implies a negative cost cycle in the residual network), therefore the number of iterations in the above described Min-Cost Flow algorithm is |*M*^∗^| ≤ *n* (where *M*^∗^ is an optimal matching of minimum size), and so the total running time of the algorithm is *O*(|*M*^∗^|*n*^2^ + *nm*), which is faster than *O*(*n*^**3**^ + *nm*) in the case where |*M*^**∗**^| is small. Again, for the RNA tree alignment application, in most cases it is expected that the matching would be computed with respect to dissimilar sets of subtrees, for which it is expected to have a small optimal matching size.

### Efficient algorithms for *All-Cavity-MCM* and *All-Pairs-Cavity-MCM*

We now present Algorithm 2, which solves *All*-*Pairs*-*Cavity*-*MCM*. Similarly to Kao et al. [28], we show that solutions for instances of the form (*X* ∖ {*x*},*Y* ∖ {*y*},*w*) correspond to certain shortest paths in the residual flow network obtained when solving the instance (*X*,*Y*,*w*). This observation allows to solve both *All*-*Cavity*-*MCM* and *All*-*Pairs*-*Cavity*-*MCM* at the same time complexity *O*(*n*^3^ + *nm*) as that of the algorithm for *MCM* presented in the previous section.

#### **Algorithm 2:** *All*-*Pairs*-*Cavity*-*MCM* (*X*,*Y*,*w*)

In order to prove the algorithm’s correctness, we first formulate an auxiliary lemma.

#### 

**Lemma 8.** Let *G* = (*V*,*E*) be a directed graph with a cost function *cost*(·) over its edges and no negative cost cycle. Let *P* be a minimum cost path from a node *y* to a node *x* in *G*. Let *G*′ = (*V*,*E*′) be the graph obtained from *G* by adding, for every edge (*u*,*v*)∈*P*, the reversed edge (*v*,*u*) (if not already in *G*), and setting its cost to *cost*(*v*,*u*) = - *cost*(*u*,*v*). Then, *G*′contains no negative cost cycle.

#### 

*Proof.* Following [30], we call a flow *extreme* if it is of minimum cost among all flows of the same size. By [30] (Theorem 3), a flow is extreme if and only if the corresponding residual network contains no negative cost cycle. In addition, a flow obtained by increasing an extreme flow along a minimum cost augmentation path from the source to the sink of the network, is also extreme (see [44], page 121).

Let *N* be the flow network defined by the graph *G*, the source node *y*, the sink node *x*, the cost function *cost*(·), and the capacity function *c* which assigns two capacity units to all edges in *G*. Since there is no negative cost cycle in *N*, the zero flow is extreme with respect to zero-size flows. Let *f* be the unit flow along *P* in *N*, and note that the residual graph *G*^*f*^ is identical to *G*′ by definition. Since *P* is a minimum cost path from *y* to *x* in *G*, *f* is extreme with respect to all flows of size 1. Thus, *G*^*f*^ contains no negative cost cycle. □

In the remainder of this section, let *N* be the network corresponding to the matching instance (*X*,*Y*,*w*) and *f* an optimal flow in *N*. Denote by *N*_*x*,*y*_ the network whose graph *G*_*x*,*y*_ is obtained by excluding from *G* the node *x*, the node *y* in case that *y* ∈ *Y*_*X*_, and all edges adjacent to these nodes. The edge costs in *G*_*x*,*y*_ are inherited from *G*, and the maximum flow size in *N*_*x*,*y*_ is *n* - 1. Note that any flow in *N* which does not transmit flow units through *x* and through *y* (when *y* ∈ *Y*_*X*_), is also a valid flow in *N*_*x*,*y*_. In addition, observe that *G*_*x*,*y*_ contains the graph corresponding to the sub-instance (*X* ∖ {*x*},*Y* ∖ {*y*},*w*) (it is possible that *G*_*x*,*y*_ contains additional nodes in *Y* ∖ {*y*} excluded from (*Y* ∖ {*y*})_*X* ∖ {*x*}_ and additional edges, since *x*′∈*X* ∖ {*x*} may have *n* adjacent edges of the form (*x*′,*y*′) in *G*_*x*,*y*_, while in the graph corresponding to (*X* ∖ {*x*},*Y* ∖ {*y*},*w*) is has (*n* - 1) such adjacent edges). Nevertheless, it can be asserted from the lemmas in Section ‘Reducing *MCM* to *Min-Cost Max-Flow*’, Proposition 1, and their proofs, that for an optimal flow *f*′ in *N*_*x*,*y*_, *MCM*(*X* ∖ {*x*},*Y* ∖ {*y*},*w*) = *cost*(*f*^′^) + *w*_*X* ∖ {*x*},*Y* ∖ {*y*}_. The correctness of Algorithm 2 is implied by the following analysis.

#### 

**Lemma 9.** For every *x* ∈ *X* and every *y* ∈ *Y*_*X*_, there is a path from *y* to *x* in *G*^*f*^.

#### 

*Proof.* If *f*(*y*,*t*) = 0, then *G*^*f*^ contains the edge (*y*,*t*), and in particular there is a path from *y* to *t* in *G*^*f*^. Else, *f*(*y*,*t*) = 1, and from flow conservations constraints there is some *x*′ ∈ *X* such that *f*(*x*′,*y*) = 1. In this case, the path *y* → *x*′ → *ϕ* → *t* is a path from *y* to *t* in *G*^*f*^ (again, the existence of all edges of this path in *G*^*f*^ can be asserted from the flow conservation constraints). Similarly, let *z*∈*Y*_*X*_ ∪ {*ϕ*} be the node such that *f*(*x*,*z*) = 1, and note that the path *t* → *z* → *x* is a path from *t* to *x* in *G*^*f*^. The concatenation of a path from *y* to *t* and a path from *t* to *x* in *G*^*f*^ proves that there is a path from *y* to *x* in *G*^*f*^. □

Now, we show the relation between *MCM* solutions for instances (*X*,*Y*,*w*) and solutions for sub-instances of the form (*X* ∖ {*x*},*Y* ∖ {*y*},*w*). As defined in Algorithm 2, let$d_{y,x}=\left\{\begin{array}{cc} d_{y,x}^{f}, & y\in Y_{X},\\ d_{t,x}^{f}, & y\in Y\setminus Y_{X} \end{array}\right)$

#### 

**Lemma 10.** *The cost of an optimal flow inN*_*x*,*y*_is *cost*(*f*)+*d*_*y*,*x*_.

#### 

*Proof.* In order to prove the lemma, we construct a flow *f*′ in *N*_*x*,*y*_ such that *w*(*f*^′^) = *cost*(*f*) + *d*_*y*,*x*_, and prove that *f*′ is optimal with respect to *N*_*x*,*y*_.

Consider the case where *y* ∈ *Y*_*X*_, and so$d_{y,x}=d_{y,x}^{f}$. From Lemma 9, there exists a path from *y* to *x* in the residual graph *G*^*f*^. Let *P* be such a path of minimum cost, and define the weighted graph *G*′ that includes all nodes and edges in *G*^*f*^ with the same edge costs, and in addition, for each (*u*,*v*) ∈ *P*, *G*′ contains the reversed edge (*v*,*u*), whose cost is set to *cost*(*v*,*u*) = - *cost*(*u*,*v*). Since *G*^*f*^ contains no negative cost cycle (due to the optimality of *f*), Lemma 8 indicates that *G*′ contains no negative cost cycle.

Define the flow *f*′ is obtained from *f* by returning one flow unit from *t* to *s* along the path *P*′ as follows: if *P* starts with (*y*,*t*), then *P*′ is obtained by removing (*y*,*t*) from *P* and concatenating (*x*,*s*) at its end (see Figure 8d). Else, *P*′ is obtained by concatenating (*t*,*y*), *P*, and (*x*,*s*). Observe that *f*′ is a valid flow in *N*_*x*,*y*_ (since *f*′ passes no flow units through *x* and *y*), its cost is *cost*(*f*′) = *cost*(*f*)+*cost*(*P*′) = *cost*(*f*) + *d*_*y*,*x*_ (since edges adjacent to *t* and *s* have zero costs, and so the costs of *P*′ and *P* are equal), and its size is |*f*′| = |*f*| - 1 = *n* - 1. Therefore, *f*′ is a maximum flow in *N*_*x*,*y*_. Also, observe that the residual graph$G_{x,y}^{f^{\prime }}$ is a sub-graph of *G*′ (since the only edges in *P*′ which are not in *P* are adjacent to either *x* or *y*, and therefore their reversed edges, which are included in$G^{f^{\prime }}$, are excluded from$G_{x,y}^{f^{\prime }}$), and therefore it contains no negative cycle. Thus, *f*′ is an optimal flow in *N*_*x*,*y*_ of cost *cost*(*f*)+*d*_*y*,*x*_.

For the case where *y* ∈ *Y* ∖ *Y*_*X*_ (and$d_{y,x}=d_{t,x}^{f}$), let *P* be a minimum cost path from *t* to *x* in *G*^*f*^, and *P*^′^ the path from *t* to *s* in *G*^*f*^ obtained by concatenating the residual edge (*x*,*s*) at the end of *P*. Similarly as above, it can be shown that the flow *f*′ obtained by returning one flow unit from *t* to *s* along *P*^′^ is an optimal flow in *N*_*x*,*y*_ of cost *cost*(*f*) + *d*_*y*,*x*_, and the lemma follows. □

From Lemma 10, for every *x* ∈ *X* and every *y* ∈ *Y*, the value *cost*(*f*) + *d*_*y*,*x*_ is the cost of an optimal flow in *N*_*x*,*y*_. From Proposition 1, adding to this cost the value *w*_*X* ∖ {*x*},*Y* ∖ {*y*}_ = *w*_*X*,*Y*_ - *w*(*x*) - *w*(*y*) gives the minimum matching cost for the matching instance (*X* ∖ {*x*},*Y* ∖ {*y*},*w*), and thus the correctness of Algorithm 2.

In order to use the algorithm for solving *All-Cavity-MCM*, we apply the following simple modification. Say we are interested in finding solutions to sub-instances of the form (*X*,*Y* ∖ {*y*},*w*) for every *y* ∈ *Y*. We replace the set *X* by the set *X*′ = *X*∪{*x*_*ϕ*_} for some new element *x*_*ϕ*_ ∉ *X* (and arbitrarily define *w*(*x*_*ϕ*_) = 0 and *w*(*x*_*ϕ*_,*y*) = 0 for every *y* ∈ *Y*). We then solve *All*-*Pairs*-*Cavity*-*MCM* for the instance (*X*′,*Y*,*w*), and return *MCM*(*X*′∖{*x*_*ϕ*_},*Y* ∖ {*y*}) as the solution for the instance *MCM*(*X*,*Y* ∖ {*y*}). Finding solutions for all instances (*X* ∖ {*x*},*Y*,*w*) is done symmetrically.

#### ***Time complexity of Algorithm 2***

From the analysis in Section ‘Efficient computation and time complexity’, lines 1 and 2 of the algorithm can be computed in *O*(*n*^3^ + *nm*) running time. After finding an optimal flow *f* in *N*, it is possible to compute for some *x* ∈ *X* the values$d_{v,x}^{f}$ for every *v* ∈ *V* in *O*(*n*^2^), as explained in Section ‘Efficient computation and time complexity’ (applying Lemma 7 and using a reversed variant of Dijkstra’s algorithm [42]). Thus, the computation of such values for all *x* ∈ *X*, performed in line 3 of the algorithm, can be implemented in *O*(*n*^3^) running time.

Given these values, the execution of line 4 takes *O*(*nm*) time. In all, the running time of Algorithm 2 is *O*(*n*^3^ + *nm*), proving the statements regarding *All*-*Cavity*-*MCM* and *All*-*Pairs*-*Cavity*-*MCM* in Theorem 1.

## Implementation details

Our algorithm is implemented (in Java) as a tool called FRUUT (Fast RNA Unordered Unrooted Tree mapper). The RNA tree representation is described in Section ‘RNA tree representation’ and the scoring scheme employed by FRUUT is described in Section ‘Alignment cost function’. FRUUT allows the user to select any alignment mode combination (rooted / unrooted, ordered / unordered, local / global) and to compute optimal pairwise alignments of RNA trees with an attached significance scoring model described in Section ‘p-Value computation’. We also provide an interactive PHP web-server for running FRUUT in our website (RNA plots are are generated by the Vienna Package [2]).

### RNA tree representation

**Figure 9:**
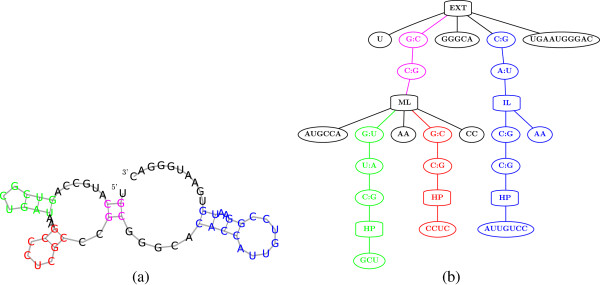
**Demonstration of the selected RNA representation.** (**a**) RNA secondary structure as presented by [2]. (**b**) The tree representation of (a) in our model. Each base-pairs stacking is matched by color to it’s representing branch in the tree.

There are several previous models for representing a pseudoknot-free RNA secondary structure (example in Figure 9a) as an ordered, rooted tree [3,9,10,13,45-48]. For example, [45] represented the RNA structure as a tree, where nodes correspond to loop elements of the secondary structure (hairpin loops, bulges, internal loops or multi-loops) and the edges correspond to base-paired (stem) regions. Another, different representation is given in Zhang’s work [9]: the nodes of the tree represent either unpaired bases (leaves) or paired bases (internal nodes). Each node is labeled with a base or a pair of bases, respectively. There are two kinds of edges, alternatively connecting either consecutive stem base-pairs or a leaf base with the last base-pair in the corresponding stem. The aforementioned trees are rooted and ordered, their order corresponds to the 5’-3’ orientation of an RNA sequence and their root is traditionally a designated node parenting the motif in which the first 5’ base participates.

The tree representation that we employed uses a similar modeling as in Höchsman et al. [48], with some variations we describe next. Given an RNA secondary structure, each loop, base-pair, and interval of unpaired bases generates a node in the tree representation of the structure. Labels are assigned to tree nodes in order to indicate their type and content. Node types are: BP (base-pair), UPI (unpaired-base interval), HP (hairpin), IL (internal loop or bulge), ML (multi-loop), and EXT (external loop). For a UPI node, the label also includes the 5’ to 3’ base-sequence of the corresponding interval, and for a BP node the label includes the corresponding bases (see Figure 9).

Each loop node (HP, IL, ML, and EXT) is connected, in 5’ to 3’ sequence order, to all UPI nodes which correspond to intervals of unpaired bases associated to the loop, and to all BP nodes which correspond to stem-terminating base-pairs adjacent to the loop. BP nodes are nodes of degree 2, where the two neighbors of such nodes are the BP nodes that correspond to adjacent stacked base-pairs. The set of leaves in the tree corresponds to the set of UPI nodes or BP nodes terminating loop-free stems.

### Alignment cost function

Node smoothing costs were set to 3 for BP, 11 for ML/EXT and 5 for IL. For subtree pruning costs, we have designed a cost function that counts the occurrences of different types of elements appearing in the subtree and deduces a corresponding penalty. The function is of the form: *prune*(*T*) = *C*_*up*_ · *N*_*up*_ + *C*_*bp*_ · *N*_*bp*_ + *C*_*hp*_ · *N*_*hp*_ + …, where the values of *C*_*x*_ are constant penalty factors, *N*_*up*_ is the number of unpaired bases in *T*, *N*_*bp*_ is the number of base-pairs, *N*_*hp*_ is the number of hairpins, and so on (specific *C*_*x*_ values are given in Table 1). Table 2 summarizes the costs of matching node pairs by the alignment. Sequence alignment costs and base-pair alignment costs were set using the RIBOSUM85-60 scoring matrix [49]. In order to support the local alignment mode, we added an option to set the subtree pruning cost to zero: *prune*(*T*) = 0.

**Table 1:** Cost functions: Element pruning penalty

**Factor**	***C***_***up***_	***C***_***bp***_	***C***_***hp***_	***C***_***ml***_	***C***_***il***_	***C***_***ext***_
**Value**	1	2.5	5	3	2	0

**Table 2:** Cost functions: Node matching costs

**Type**	**UPI**	**BP**	**HP**	**IL**	**ML**	**EXT**	
UPI	Sequence alignment	*∞*	*∞*	*∞*	*∞*	*∞*	
BP	*∞*	Base-pair alignment	*∞*	*∞*	*∞*	*∞*	
HP	*∞*	*∞*	-10	0	0	0	
IL	*∞*	*∞*	0	-5	*∞*	*∞*	
ML	*∞*	*∞*	0	*∞*	-7	-7	
EXT	*∞*	*∞*	0	*∞*	-7	-10	

### Relative scoring

We used a relative score formula described by Höchsmann et al. [48] to assess the similarity of two trees, normalizing the alignment cost by the average of the self-alignment costs of the compared trees. Let *HSA*_*m*_(*T*,*S*) denote the optimal alignment cost of trees *T* and *S* in alignment mode *m*, where *m* is one of the following modes: *Rooted-Ordered*, *Rooted-Unordered*, *Unrooted-Ordered* or *Unrooted-Unordered*. Let *RelScore*_*m*_(*T*,*S*) denote the relative score of *T* and *S* in alignment mode *m*, given by [48]:

(8) $$\text{RelScor}e_{m}(T,S)=\frac{2\text{HS}A_{m}(T,S)}{\text{HS}A_{m}(T,T)+\text{HS}A_{m}(S,S)}\;.$$

The scoring scheme we use satisfies that for every tree *T*, *HSA*_*m*_(*T*,*T*) < 0, and for every pair of trees *T*,*S*, *HSA*_*m*_(*T*,*T*),*HSA*_*m*_(*S*,*S*) ≤ *HSA*_*m*_(*T*,*S*). Under these conditions, the relative score for any pair of trees is upper bounded by 1, and the similarity of the trees increases as the score approaches 1.

### p-Value computation

We apply the following *p-Value* computation in order to determine whether an alignment score obtained by comparing two RNA trees is significant.

**Figure 10:**
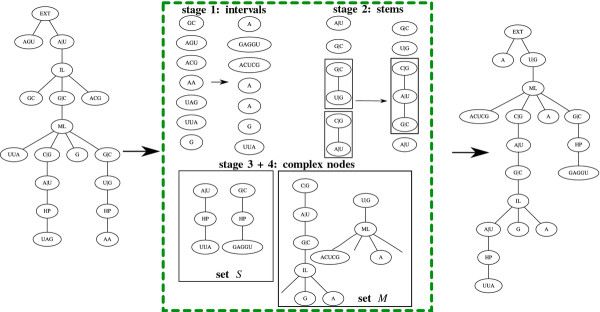
**Example of the shuffle process.** On the left is the input tree and on the right the output of the process.

For the purpose of assessing the significance of a score, we need to know what distribution *HSA* scores follow. In order to identify the correct distribution, we first create a set of random observations to inspect. Algorithm 3 describes a routine for shuffling a tree, while preserving most of its structural features (e.g. number of stacks, amount of multi-loops) and keeping the tree valid in terms of RNA secondary structure (exemplified in Figure 10).

#### **Algorithm 3:** shuffle (*T*)

Algorithm 4 creates a set of observations by randomly selecting pairs of trees from a dataset, shuffling them and reporting the relative score of their alignment. In each alignment mode *m*, where *m* ∈ {*RO*,*RU*,*UO*,*UU*}, we ran Algorithm 4, setting the input parameter *dataset* to all the structures from over the RNAStrand [50] database (containing 1751 RNA structures), and setting the number of iterations (the *amount* parameter, in the code below) to 2 × 10^6^.

#### Stats (*dataset*,*amount*)

**Figure 11:**
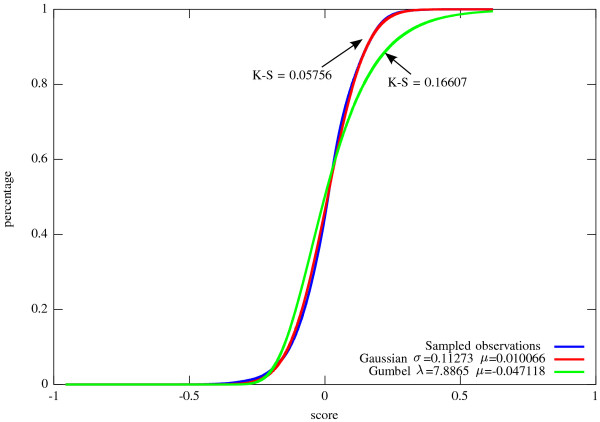
**The result of running*Stats***(***D***,1 × 10^6^) in***UU*** mode of***HSA*** with relative score and its fitting to Gaussian distribution.

The observed results where plotted in an accumulative manner and a Maximum Likelihood (ML) fitting technique was used to determine the best distribution and its parameters. We tried several types of distributions and used the Kolmogorov-Smirnov test (K-S test) formula [51] to measure the goodness of the data fit to a given distribution. In all four alignment modes the ML Gaussian distribution provided the best fit. Figure 11 exemplifies the fitting of the data to this distribution with alignment mode *UU*. The figure also displays the ML Gumbel distribution. The K-S score of the ML Gaussian distribution is better than the score for the ML Gumbel distribution. Following these results we used the ML fitted Gaussian distribution to model the HSA results. This allowed us to compute for a pair of trees *T* and *S* a p-Value score analytically, using the following formula:

p-value(x)=Pr(X>x),

where *x* is the relative score of *T* and *S*, and *X* is a random variable normally distributed with the ML fitted parameters.

A Bonferroni correction was applied to all the reported p-value computations described in the following sections. This was done by multiplying the computed p-value by the number of tests performed (i.e. the number of tree pairs aligned within the family that participated in the corresponding test).

#### **Algorithm 4:** Stats (*dataset, amount*)

## Results

### RNase P family

RNase P is the endoribonuclease responsible for the 5’ maturation of tRNA precursors [19]. Secondary structures of bacterial RNase P RNAs have been studied in detail, primarily using comparative methods [52], and were shown to share a common core of primary and secondary structure. In bacteria, synthetic minimal RNase P RNAs consisting only of these core sequences and structures were shown to be catalytically proficient. Sequences encoding RNase P RNAs of various genomes have been determined and a database established [53], which consists of a compilation of ribonuclease P RNA sequences, sequence alignments, secondary structures and three-dimensional models.

We conducted a preliminary experiment, intended to identify examples of pairs of RNA trees for which an RNA structural comparison approach supporting unrooting and branch shuffling may detect (otherwise hidden) structural similarity. To achieve this, we ran a benchmark of all-against-all pairwise alignments of bacterial RNAse P RNA secondary structure trees, using our tool’s different tree-alignment modes and comparing the differences between the obtained alignment costs. The alignment cost functions and parameters used in our experiment are given in Section ‘Alignment cost function’.

Our benchmark was based on 470 RNAse P structures, ranging across various organisms, taken from the RNAse P database [53] (molecule naming conventions are according to [50]). After filtering out partial and environmental sequences, 170 distinct structures remained, yielding 14,365 distinct pairs of trees. The sizes of the trees in this dataset ranged from 82 to 230 nodes, averaging at 142. The total running time of the benchmark was approximately 33 minutes on a single Xeon X5690 using around 300Mb of memory.

Each pair of trees *T*,*S* was compared in two modes to obtain the corresponding scores and alignments: rooted-ordered (RO) and rooted-unordered (RU), and the relative score was computed for each pair in each mode according to Section ‘Relative scoring’.

Our goal in this experiment was to identify evolutionary events that can be explained by unordered alignments. Thus, we sought pairs of RNAse P RNAs that are highly conserved, and yet their alignment can still benefit substantially from unordered mappings. To achieve this, we removed from the set pairs of trees for which *RelScore*_*RU*_(*T*,*S*) < 0.5. We sorted the remaining pairs of trees according to the difference between the *RU* and *RO* modes (*RelScore*_*RU*_(*T*,*S*) - *RelScore*_*RO*_(*T*,*S*)).

When examining the top 50 alignments carefully, two distinct types of mapping patterns were observed among them, where each of the top 50 pairs belongs (with slight variations) to one of these two types (33 to Type 1 and 17 to Type 2). In the next paragraphs, we exemplify the highest ranking alignment of each of the two types (the first type is shown in Figure 1b). As mentioned before, the input for FRUUT alignments consisted only of sequence and secondary structure information. The tertiary structure (pseudoknot annotations) for the top-ranking alignments were only considered later, during the alignment interpretations.

**Figure 12:**
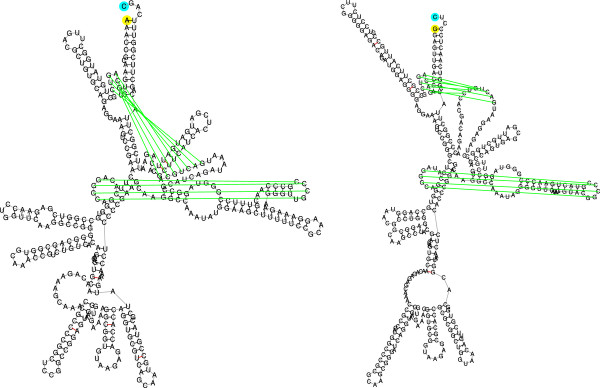
**RNAse P type 1 Tertiary structural information.** Tertiary structural information for *ASE_00047* and *ASE_00334*, taken from the RNAse P Database [53].

*Type 1: loop swapping in main multiloop accompanied by hairpin deletion in P17.1* The first type of alignment pattern is characterized by comparisons between a green sulfur bacteria *Chlorobium* and gamma purple bacterial RNAse P RNAs. This alignment pattern is exemplified in Figure 1b, for the bacterial RNAs *ASE_00047* and *ASE_00334*. When examining the corresponding tertiary structure information (Figure 12), the transformations predicted by FRUUT seem to make sense: observe that there is an additional duplex connecting the loops of intervals 13 and 15. Notice that the alignment between the two trees maps the intervals in a manner that preserves the tertiary structural information and the other information surrounding the loops - thus exemplifies a biologically verified alignment which does not preserve branch ordering.

**Figure 13:**
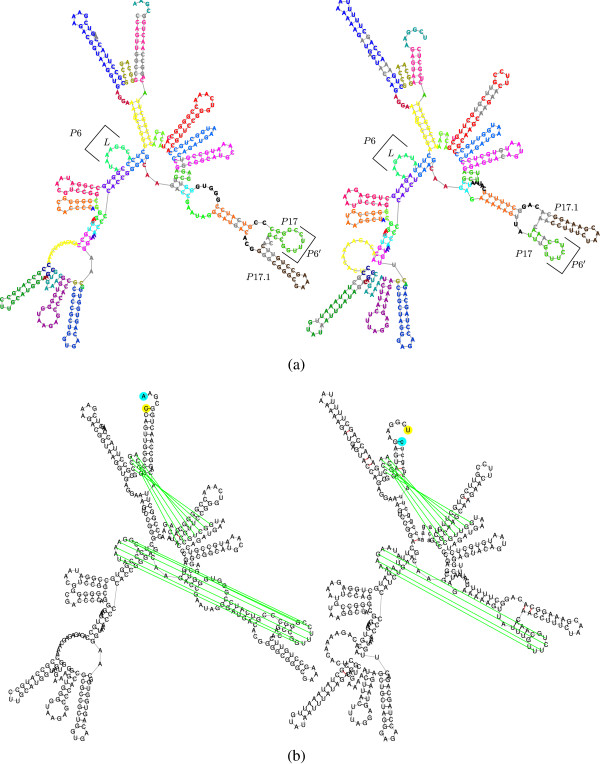
**RNAse P type 2 example.** (**a**) An example of unordered alignment between two RNAse P RNAs: *ASE_00018* and *ASE_00070* with a corrected p-value score of 3.117×10^-6^. Grey colored bases in the FRUUT alignment graphics represent deletions. *P*6 is a pseudoknot marking of the tertiary structure information. (**b**) Tertiary structural information for *ASE*_00018 and *ASE*_00070, taken from the RNAse P Database [53].

*T*ype 2: hairpin swapping between P17 and P17.1 The second type of alignment pattern is characterized by comparisons between *Agrobacterium tumefaciens* (*ASE_00018*) and several *Chlamydia trachomatis* members. This alignment pattern is exemplified in Figure 13. An interesting element-twist transformation is observed here in hairpins *P*17 and *P*17.1 of *ASE_00018*, which are mapped onto their corresponding hairpins *P*17.1 and *P*17 in *ASE_00070*, respectively, via a subtree reordering mapping operation. When examining the corresponding tertiary structure information (Figure 13), we observe that the loops of the hairpins *P*17 and *P*17.1 are engaged in pseudoknots with loops *L* (named P6).

### The Hammerhead Ribozyme family

Another type of homology detected by our tool is exemplified in the Hammerhead Ribozyme family, which is characterized by two distinct transcript types, yielding the same functional RNA (Figures 1a and14).

**Figure 14:**
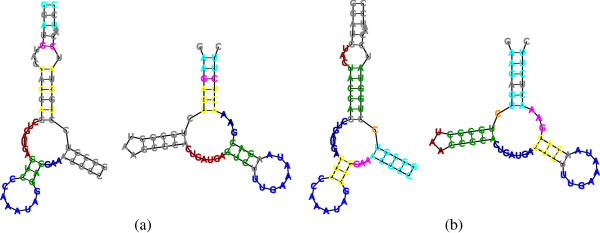
**Example 2 on the HammerHead family.** Results on different modes of alignment between two Hammerhead RNAs: *PDB_01077* (Type I, left) and *RFA_00433* (Type III, right) (**a**) A rooted ordered alignment between the two trees with a relative score of 0.2374. (**b**) A unrooted ordered alignment between the two trees with a relative score of 0.5111.

The Hammerhead Ribozyme, a derivative of several self-cleaving satellite virus RNAs [54], is a single strand RNA with autocatalytic capabilities. Naturally, it has a highly specific self-cleavage site at *C17*, operating via isomeration and rearrangement of the linkage phosphodiester bond [55]. Furthermore, Birikh et al.[18] suggested that the Hammerhead Ribozyme may undergo synthetic modification by removing the loop of one of its helical arms, thus making it catalytically active and able to cleave other RNAs. Hammerheads are therefore widely used in the biotechnological industry as biosensors, enzymes for specific RNAs and gene discovery agents.

The tertiary structure of the minimal version of the Hammerhead Ribozyme has been thoroughly studied by [56,57]. It is composed of three base paired helices, entitled I, II, III, according to their position in the sequence. There are highly conserved sequences within the multi-loop between stem I and II (containing the sequence box *CUGA*), between stem II and III (containing the box *GAAA*) and between stem III and I (containing the cleavage reaction site, *C17*). In nature, there are two types of Hammerheads: type I, where stem I starts at the 5’ and 3’ ends of the strand, and type III, where stem III starts at the 5’ and 3’ ends of the strand. As of today, no natural type II Hammerhead has been found.

Due to the fact that the two natural Hammerhead types share a conserved structure, though it is formed by two distinct transcripts, we chose this family to demonstrate the sensitivity gained by extending the classical rooted-ordered RNA tree alignment to more flexible variants. Our benchmark was based on 146 Hammerhead structures (86 of type *I* and 62 of type *III*), ranging across various organisms, taken from the RNA Strand database [50]. This yields a total of 10,585 pairs of trees, with tree sizes ranging from 17 to 48 nodes, averaging at 25.5.

Each pair of trees *T*,*S* was compared in two modes to obtain the corresponding scores and alignments: rooted-ordered (RO) and unrooted-ordered (UO). Within each mode, we used the relative score formula as described in Section ‘Relative scoring’. We partitioned the tree pairs into two groups, the first containing all pairs where both members are from the same type (5377 pairs) and the second group containing mixed pairs where the two members in each pair belong to different types (5208 pairs). We calculated the average relative score for each group in both alignment modes. The results, summarized in Table 3, show that the UO tree alignment mode is more sensitive to the similarity between the two different Hammerhead types than the RO mode. The similarity between Hammerhead structures of different types is not captured by the classical rooted ordered alignment (Figure 14a). However, when comparing the same pair using unrooted ordered alignment, the similarity between the structures is revealed, in line with the similarity of biological function (Figure 14b).

**Table 3:** **A comparison of the *RO* and*UO* alignment modes**

**Mode ∖ Type**	**Same type**	**Different type**
Rooted-Ordered (**RO**)	0.55	0.19
Unrooted-Ordered (**UO**)	0.56	0.35

A comparison of the *RO* and *UO* alignment modes on the two types of Hammerhead structures. Each cell in the table represents the average over all pairs in each group (same / different types).

## Conclusions

In this paper we define the (unrooted unordered) Homeomorphic Subtree Alignment (HSA) problem, as well as additional three restricted variants of it: Ordered-HSA, Rooted-HSA, and Ordered-Rooted-HSA. We focus on the general (unrooted and unordered) HSA variant, and present a cubic time algorithm for it.

The new algorithm is implemented as a tool (which allows solving all four HSA variants) and is applied to pairwise alignments of RNA secondary structures. Preliminary experimental results over members of the RNAs P and Hammerhead families show that the tool can be used for detecting new structural similarities between RNA molecules, which could not be detected by the classical rooted-ordered tree alignment methods. In order to obtain an *O*(*n*^3^) running time of an otherwise *O*(*n*^5^) time algorithm, we extend the *All*-*Cavity* Bipartite Matching problem, previously defined by Kao et al., to the *All*-*Pairs*-*Cavity* problem, give an efficient algorithm for it, and show how to integrate it as a subroutine within our tree alignment algorithm.

## Competing interests

The authors declare that they have no competing interests.

## Authors’ contributions

NM participated in some parts of the algorithm construction, implemented the software, participated in the writing of the manuscript and was in charge of the data collection, the statistical model, the experimental results, and the web tool. SZ contributed in all parts of the algorithm construction, participated in the software implementation and in the writing of the manuscript. EK participated in a preliminary version of this study. EB participated in the statistical model and in the experimental results. YD participated in some parts of the algorithm construction and in the overall checking of the text. MZU conceived, designed and led the study, and participated in all parts of the project, including the algorithm construction, the experimental results and the writing of the manuscript. All authors read and approved the final manuscript.
